# Mutation profiling of cancer drivers in Brazilian colorectal cancer

**DOI:** 10.1038/s41598-019-49611-1

**Published:** 2019-09-23

**Authors:** Wellington dos Santos, Thais Sobanski, Ana Carolina de Carvalho, Adriane Feijó Evangelista, Marcus Matsushita, Gustavo Nóriz Berardinelli, Marco Antonio de Oliveira, Rui Manuel Reis, Denise Peixoto Guimarães

**Affiliations:** 1 Molecular Oncology Research Center, Barretos Cancer Hospital, Barretos, Brazil; 2 Department of Pathology, Barretos Cancer Hospital, Barretos, Brazil; 3 Life and Health Sciences Research Institute (ICVS), Medical School, University of Minho, Braga, 4710-057 Portugal; 4 3ICVS/3B’s-PT Government Associate Laboratory, Braga, 4710-057 Portugal; 5 Department of Endoscopy, Barretos Cancer Hospital, Barretos, Brazil

**Keywords:** Colorectal cancer, Cancer genetics, Oncogenesis, Cancer genomics

## Abstract

The molecular basis of colorectal cancer (CRC) can guide patient prognosis and therapy. In Brazil, knowledge on the CRC mutation landscape is limited. Here, we investigated the mutation profile of 150 cancer-related genes by next-generation sequencing and associated with microsatellite instability (MSI) and genetic ancestry in a series of 91 Brazilian CRC patients. Driver mutations were found in the *APC* (71.4%), *TP53* (56.0%), *KRAS* (52.7%), *PIK3CA* (15.4%) and *FBXW7* (10.9%) genes. Overall, genes in the MAPK/ERK, PIK3/AKT, NOTCH and receptor tyrosine kinase signaling pathways were mutated in 68.0%, 23.1%, 16.5%, and 15.3% of patients, respectively. MSI was found in 13.3% of tumors, most of which were proximal (52.4%, *P*< 0.001) and had a high mutation burden. European genetic ancestry was predominant (median of 83.1%), followed by Native American (4.1%), Asian (3.4%) and African (3.2%). *NF1* and *BRAF* mutations were associated with African ancestry, while *TP53* and *PIK3CA* mutations were inversely correlated with Native American ancestry. Our study suggests that Brazilian CRC patients exhibit a mutation profile similar to other populations and identify the most frequently mutated genes, which could be useful in future target therapies and molecular cancer screening strategies.

## Introduction

Colorectal cancer (CRC) is the third most common type of cancer worldwide^1,2^. According to GLOBOCAN, over 1.8 million new colorectal cancer cases occurred in 2018. Moreover, the disease is the second cause of death by cancer worldwide^2^. Increases in both incidence and mortality have been observed over the last 10 years in several Europe, Latin America, and Asia countries^1^. The most significant increases in incidence were observed in Brazil, Costa Rica and Bulgaria^1^. In Brazil, the Brazilian National Cancer Institute (INCA) estimated that over 36,000 new cases were expected for 2018, ranking CRC as the third most frequent cancer among men and as the second most frequent cancer among women^3^.

CRC is more frequently observed in the distal colon (left colon, from splenic flexure to rectum) than in the proximal side (right colon, from the cecum to transverse colon)^4^. In addition to incidence differences, the tumors arising from the left and right colon are distinct in their epidemiology, biology, histology and microbial diversity^4–7^. Consequently, it also influences patients’ prognosis^4,8^.

Several lifestyles are risk factors associated with CRC, such as red and processed meat consumption, alcohol intake, smoking and body weight^9^. Moreover, the cumulative acquisition of genetic alterations leads to a progressive tumorigenesis process from normal to precursor lesion, culminating in a malignant tumor^10^. The majority (80–85%) of CRC cases are sporadic and emerge from somatic alterations in driver genes^10^. These alterations are linked to three main molecular groups: chromosomal instability, mismatch repair defect and methylator phenotype^7,10^. In addition to these classic features, the CRC Subtyping Consortium classified four consensus molecular subtypes (CMS) of CRC: CMS1, which are enriched for tumors with MSI, overexpression of DNA damage repair proteins, high immune activation and widespread hypermethylation; CMS2, which have tumors with chromosomal instability and activation of the WNT and MYC signaling pathways; CMS3, with tumors with epithelial and metabolic dysregulation; and CMS4, which have tumors with transforming growth factor-β activation, stromal infiltration and angiogenesis^6^. The molecular profile of CRCs is distinct between proximal and distal tumors. Proximal CRCs are associated with microsatellite instability (MSI) and activating mutations on the *BRAF* gene, frequently harboring a high mutation burden, whereas distal CRCs are associated with chromosomal instability and are more frequently non-hypermutated and have less frequently the MSI phenotype^6,7^. In line with this, proximal CRCs are enriched for CMS1 tumors and, inversely, distal CRC are enriched for CMS2 tumors^6^.

Molecular characterization is crucial for CRC patient care and prognostic and therapeutic assessment^11–13^. Knowledge of the mutation profile of Brazilian CRC patients is limited. Therefore, the present study aimed to interrogate the mutational profile of CRCs by next-generation sequencing mutation analysis of a large panel of 150 cancer-related genes and to identify significant cancer drivers. Moreover, the molecular profile was associated with clinicopathological features and genetic ancestry markers of CRC patients.

## Results

### Description of the mutation profile

**Figure 1:**
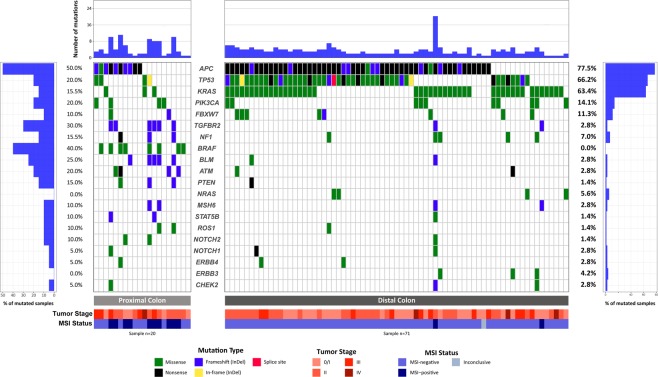
Waterfall plot (oncoplot) of the distribution of mutations found in our tumor samples. The upper plot shows the frequency of mutation for each tumor sample. Left and right plots show the frequency of samples mutated for proximal colon (left) and distal colon (right). The central plot shows the types of mutations in each tumor sample (when the sample showed more than one mutation in the same gene, only the most deleterious type is shown). The lower part of the figure shows the tumor stage at diagnosis and microsatellite instability status (MSI) of each sample. Tumor stage: stage of the disease at the diagnosis.

We sequenced 150 cancer-related genes in 91 colorectal tumors. The mean read depth of sequencing was 625.7x per gene and 60.7x per variant. Overall, after filtering out non-driver variants (see Material and Methods), we identified at least one somatic variant for each tumor sample with a mean of 3.6 mutations per patient (range 1–20). Thirteen tumors (14.3%) harbored mutations in single genes, 26 tumors (28.6%) in two genes, 24 tumors (26.4%) in three genes, and 28 tumors (30.8%) harbored alterations in four or more genes. Among the 150 genes sequenced, 46 showed at least one driver somatic mutation, including missense, frameshift, nonsense, in-frame, and splice mutations (54.9%, 22.6%, 21.3%, 0.9%, 0.3%, respectively). The most recurrently altered genes in our study population are shown in Fig. 1 (for a complete list of variants identified, see Supplementary Table S1). *APC* was the most affected gene, altered in 65 tumor samples (71.4%), followed by *TP53* (51 cases, 56.0%), *KRAS* (48 cases, 52.7%), *PIK3CA* (14 cases, 15.4%) and *FBXW7* (10 cases, 10.9%, Fig. 1). Below, we further describe the most affected genes and pathways.

### *APC* gene

A total of 86 somatic mutations in *APC* were found in 65 tumors (71.4%), among which 44 harbored a single mutation, and 21 tumors harbored two mutations in the gene. The majority of the mutations were protein truncating: 52 nonsense (60.5%), 30 frameshift (34.8%), and 4 missense (4.7%) (Supplementary Fig. S1 and Supplementary Table S1). Among the mutations found, 68 were previously reported as somatic mutations in CRC (50 nonsense and 14 frameshift), and 22 were new mutations (16 frameshift, 4 missense and 2 nonsense mutations). The mutations found are spread widely throughout the coding region of the *APC* gene, and the most affected site was the protein position 1556, which showed frameshift mutations in 6 tumors (Supplementary Fig. S1). In addition, nine *APC* mutated cases were selected and PCR-followed by direct Sanger sequencing was performed, validating all mutations (Supplementary Fig. S2).

### *TP53* gene

Fifty-six mutations on *TP53* were found affecting 51 tumors (56.0% of the tumors, Fig. 1 and Supplementary Fig. S1), among which 46 harbored a single mutation on the gene and 5 harbored two mutations. The majority of alterations in *TP53* were missense mutations (40, 71.4%), followed by nonsense (7 mutations, 12.5%), frameshift (5 mutations, 8.9%), in-frame (3 mutations, 5.4%) and splice region (1 mutation, 1.8%) (Supplementary Fig. S1 and Supplementary Table S1). Among the mutations found, 53 were previously reported as somatic mutations in CRC (40 missense, 7 nonsense, 3 frameshift, 2 in-frame, and 1 splice region mutations), and 3 were new mutations, including 2 frameshift and 1 in-frame mutation. Most mutations were localized on the DNA-binding domain (only one mutation was found outside this domain), and the most frequently altered site was protein position 273, with 6 missense mutations (Supplementary Fig. S1). In addition, nine *TP53* mutated cases were selected and PCR-followed by direct Sanger sequencing was performed, validating all mutations (Supplementary Fig S2).

### MAPK-ERK signaling pathway alterations

**Figure 2:**
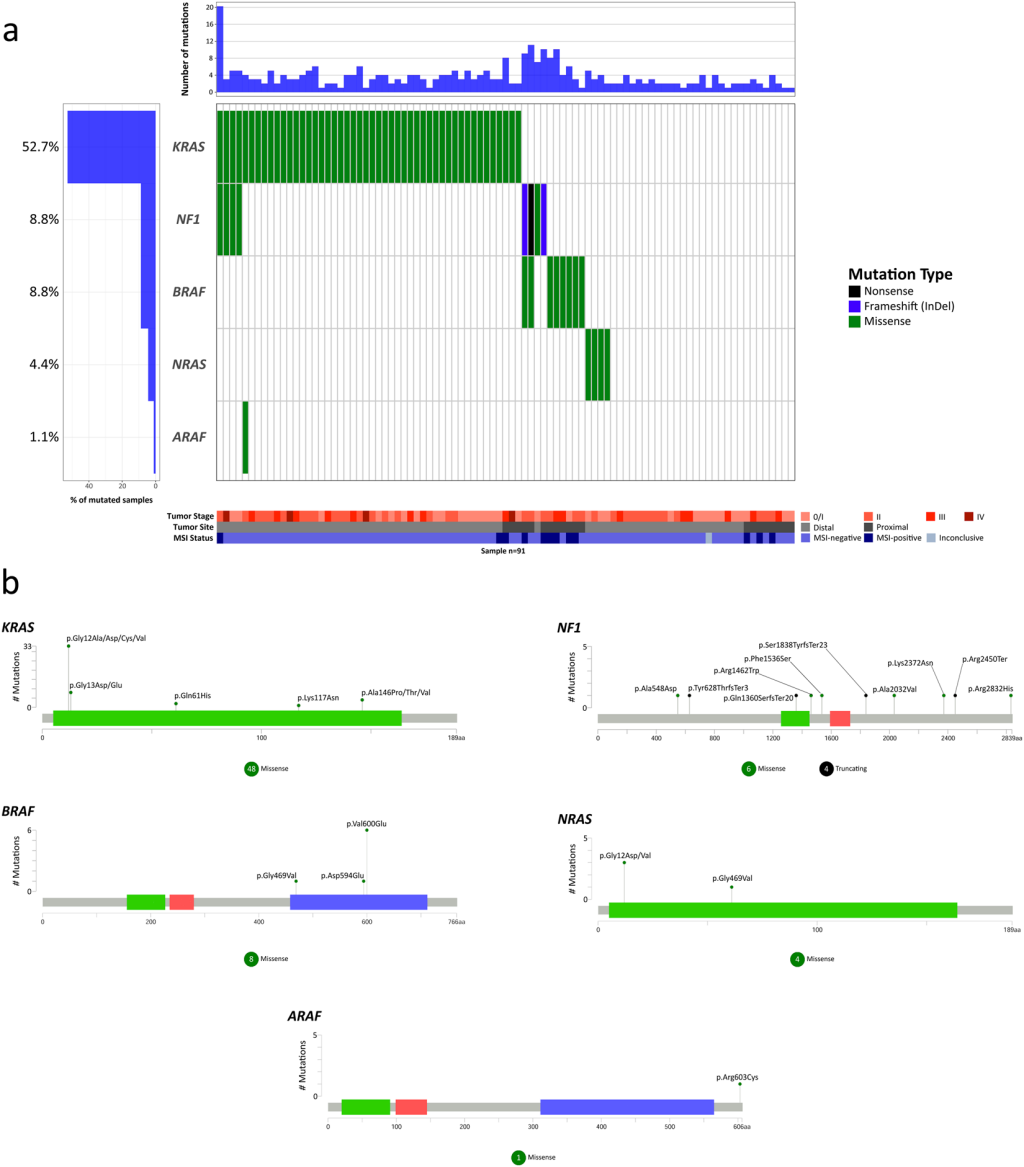
Waterfall (oncoplot) plot (**a**) and mutations mapper (**b**) of MAPK-ERK signaling pathway genes. Tumor stage: stage of the disease at diagnosis. MSI: microsatellite instability.

The MAPK-ERK signaling pathway had at least one mutated gene in 62 tumors (68%). The most frequently altered gene in the MAPK/ERK pathway was *KRAS*, which showed 48 missense mutations, affecting 52.7% of the tumors. All *KRAS* mutations affected hotspots of the gene at protein sites 12 (33 mutations, 68.7% of the *KRAS* mutations), 13 (8 mutations, 16.7%), 146 (4 mutations, 8.3%), 61 (2 mutation, 4.2%), and 117 (1 mutation, 2.1%) (Fig. 2, and Supplementary Table S1). All mutations on codons 12/13 and codon 61 of the *KRAS* gene were confirmed by the cobas^®^ *KRAS* mutation test. The *NF1* gene showed 10 somatic mutations in 8 tumor samples (8.8%), three of which were already known mutations (2 missense and 1 nonsense mutations), four were novel missense mutations, and three were novel frameshift mutations. The mutations were spread throughout the coding region of the gene. All truncating protein mutations in *NF1* were mutually exclusive with *KRAS*, and four tumors had missense mutations on both *KRAS* and *NF1* (Fig. 2a). The *BRAF* gene was mutated in 8 tumor samples (8.8%), showing 8 missense somatic mutations mutually exclusive with *KRAS*, all previously reported as somatic mutations in CRC. The p.Val600Glu mutation (p.V600E) was the most prevalent mutation, occurring in 6 tumors (75% of all mutations on *the BRAF* gene). The two variants, different from V600E mutations, also affected the kinase domain of the *BRAF* protein (p.Asp594Glu and p.Gly469Val). Mutations in the *NRAS* gene were found in 4 tumor samples (4.4%), 3 at codon 12 (75%) and one at codon 61. *NRAS* mutations were mutually exclusive with *KRAS*, *NF1* and *BRAF* mutations. Finally, the *ARAF* gene had one previously known missense mutation that was located outside of the main domains of the protein (Fig. 2 and Supplementary Table S1).

### PI3K-AKT signaling pathway alterations

**Figure 3:**
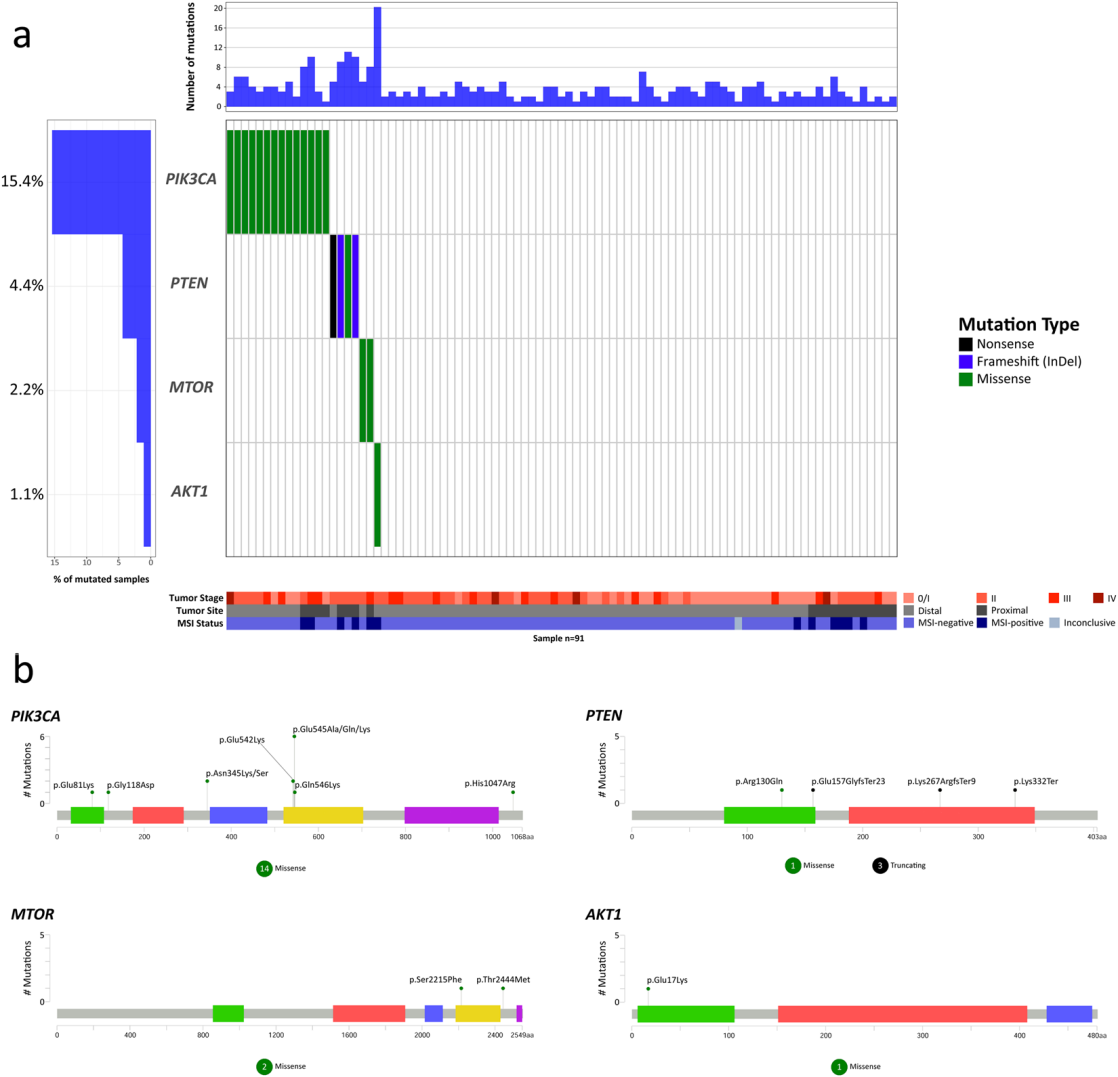
Waterfall (oncoplot) plot (**a**) and mutation mapper (**b**) of PI3K-AKT signaling pathway genes. Tumor stage: stage of the disease at diagnosis. MSI: microsatellite instability.

Genes in the PI3K-AKT signaling pathway were altered in 21 tumors (23.1%). The most frequently altered gene in the PI3K-AKT signaling pathway was *PIK3CA*, with alterations in 14 tumors (15.4%, Fig. 3a), all harboring already known somatic mutations. The PIK domain of the protein harbored most of the mutations found in *PIK3CA*, 6 mutations at codon 545 (42.9% of all *PIK3CA* mutations), 2 mutations at codon 542 (14.3%), and 1 mutation at codon 546 (7.1%). The p85-binding domain of the protein harbored 1 mutation (7.1%), and the other 4 (28.6%) mutations affected sites outside the main domains of the protein (Fig. 3a). The *PTEN* gene was mutated in 4.4% of the tumors (Fig. 3), showing one previously reported missense mutation and one frameshift mutation, along with two novel frameshift and nonsense mutations. The *MTOR* gene was mutated in 2 tumors (2.2%, Fig. 3), showing two previously described missense mutations in CRC, one affecting the PI3K/PI4K domain and one outside the main domains of the protein. *AKT1* was mutated in 1 tumor (1.1%, Fig. 3) and showed one already known missense mutation affecting the Pleckstrin homology (PH) domain of the protein.

### Notch signaling pathway alterations

**Figure 4:**
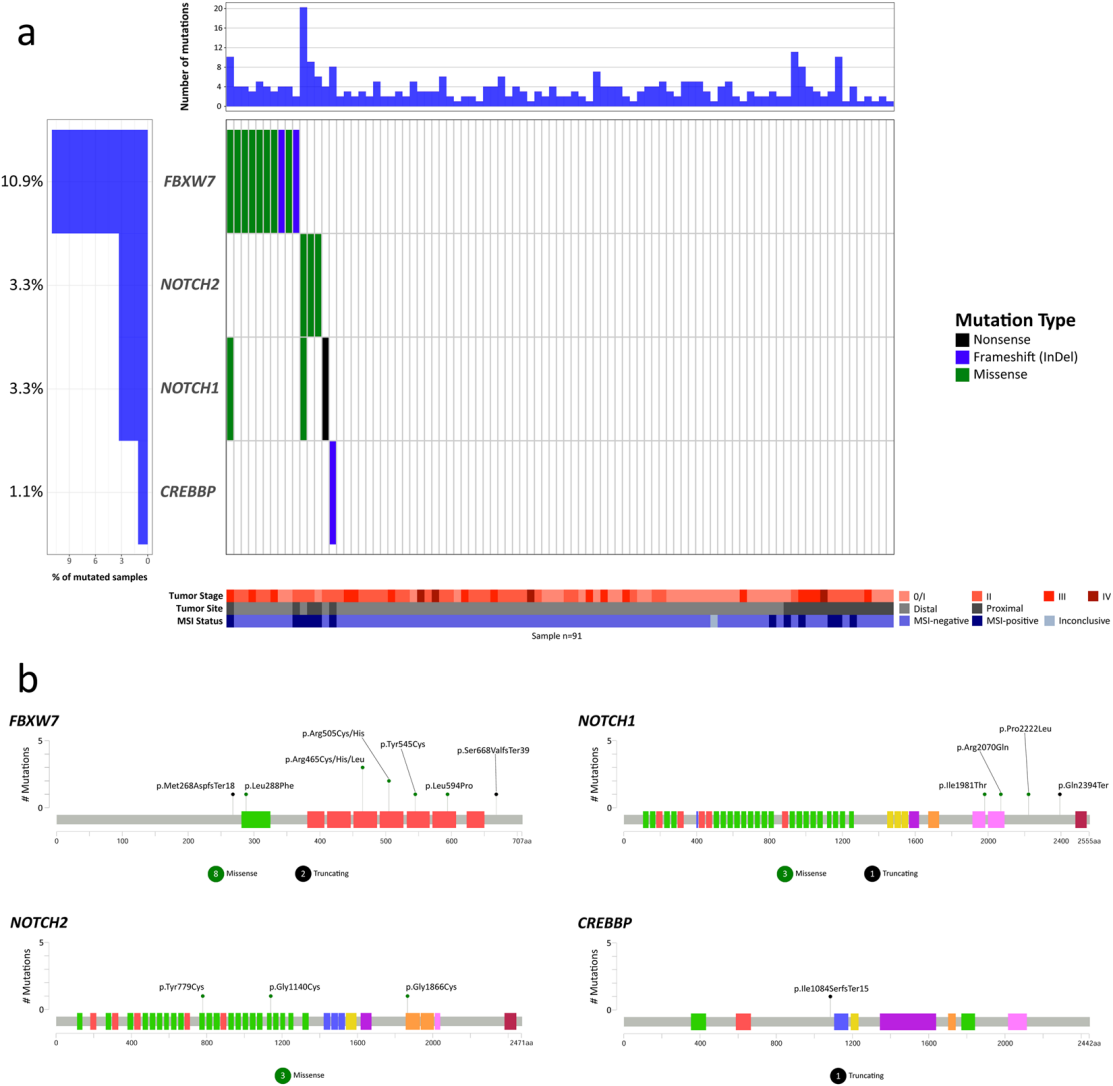
Waterfall (oncoplot) plot (**a**) and mutation mapper (**b**) of Notch signaling pathway genes. Tumor stage: stage of the disease at diagnosis. MSI: microsatellite instability.

The Notch signaling pathway was altered in 15 tumors (16.5%). *FBXW7* was the most affected gene in this pathway, mutated in 10 tumors (10.9%, Fig. 4), showing nine already known mutations (7 missense and 2 frameshift mutations) and one novel somatic missense mutation. Most of *the FBXW7* mutations (7, 70%) affected one of the repeated beta-transducin domains. One mutation (10%) affected the F-box-like domain, and 2 mutations were localized outside the main domains of the protein (Fig. 4b). The *NOTCH2* gene was altered in 3 tumors (3.3%, Fig. 4), all harboring novel missense mutations located at one of the repeats of the EGF-like domain (2 mutations) and one at the ankyrin repeat. The *NOTCH1* gene was altered in three tumors (3.3%, Fig. 4) with 4 mutations, three already known mutations (2 missense and one nonsense mutation) and one novel missense mutation. Two *NOTCH1* mutations were localized at one of the ankyrin repeats and two outside of the main domains of the protein. Finally, *the CREBBP* gene was altered in 1 tumor (1.1%, Fig. 4), showing one already known frameshift mutation localized outside the main domains of the protein.

### Receptor tyrosine kinase alterations

**Figure 5:**
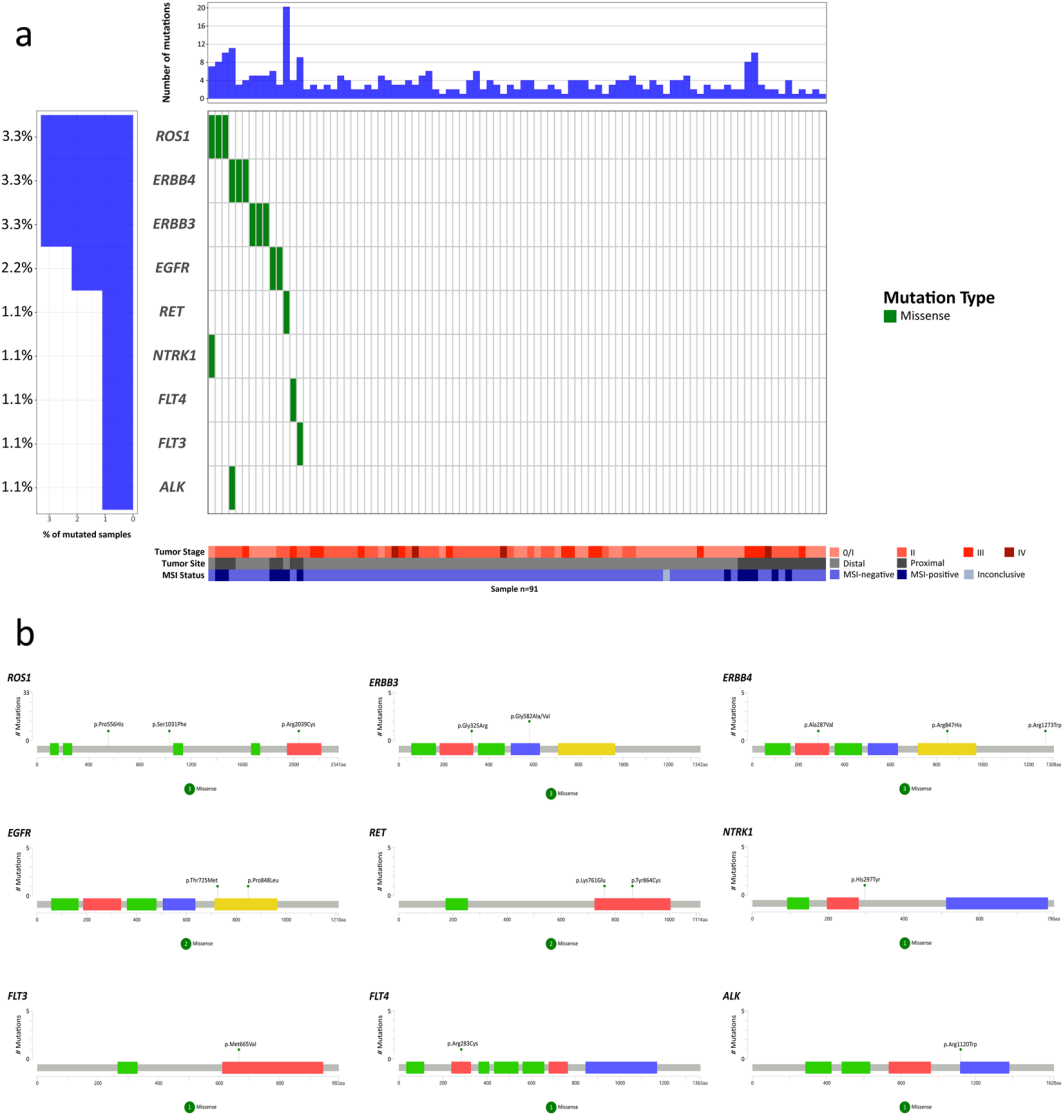
Waterfall (oncoplot) plot (**a**) and mutation mapper of RTK genes. Tumor stage: stage of the disease at diagnosis. MSI: microsatellite instability.

The receptor tyrosine kinase (RTK) family showed 9 altered genes (Fig. 5). *ROS1* was altered in 3 tumors (3.3%, Fig. 5), showing one previously described missense mutation in the protein tyrosine kinase domain and two novel missense mutations outside the main domains of the protein. The *ERBB4* gene was altered in 3 tumors (3.3%, Fig. 5), showing one novel missense mutation in the furin-like cysteine rich region of the protein, one previously described missense mutation in the protein tyrosine kinase domain, and one previously described missense mutation outside the main domains of the protein. *ERBB3* was altered in 3 tumors (3.3%, Fig. 5), showing 1 already known missense mutation in the furin-like cysteine rich region of the protein and 2 novel missense mutations in growth factor receptor domain IV. The *EGFR* gene was altered in 2 tumors (2.2%, Fig. 5), showing 2 known missense mutations in the protein tyrosine kinase domain. The *RET* gene was mutated in 1 tumor (1.1%, Fig. 5), showing two novel mutations in the protein tyrosine kinase domain. The *NTRK1* gene was mutated in 1 tumor (1.1%, Fig. 5), showing one novel missense mutation outside the main domains of the protein. The *FLT3* gene was mutated in 1 tumor (1.1%, Fig. 5), showing one novel missense mutation in the protein tyrosine kinase domain. The *FLT4* gene was altered in 1 tumor (1.1%, Fig. 5), showing one already known missense mutation in the immunoglobulin I-set domain. Finally, the *ALK* gene was also altered in 1 tumor (1.1%, Fig. 5) and showed one previously described missense mutation in the protein tyrosine kinase domain.

### MSI status and its association with tumor mutation burden

We assessed the MSI status of tumor samples by multiplex PCR. Seventy-eight did not exhibit microsatellite instability (MSI-negative, 86.7%), and twelve tumors showed microsatellite instability (MSI-positive, 13.3%). The mean mutation burden of tumors that were MSI-positive was higher than MSI-negative tumors (7.1 ± 1.4 and 3.1 ± 0.2, respectively, *P* = 0.002, Supplementary Fig. S3). Tumors with MSI-positive status also showed a higher frequency of frameshift mutations (49.4% versus 13.2% in MSI-negative tumors, *P* < 0.001) and a lower frequency of missense (43.5% vs 59.1%, *P* = 0.01) and nonsense mutations (5.9% versus 26.4%, *P* < 0.001, Supplementary Fig. S3).

The profile of somatic mutations between tumors with and without MSI was also distinct. Somatic mutations of mismatch repair (MMR) genes were found only in MSI-positive tumors, which showed four frameshift mutations on *MSH6* and one nonsense in *MLH1*. Likewise, *TGFBR2*, *NOTCH2*, and *STAT5B* genes were exclusively mutated in MSI-positive tumors (*P* < 0.001, *P* = 0.002 and *P* = 0.002, respectively, Table 1). Other genes also showed mutations only in MSI-positive tumors; however, there was no significant difference (Supplementary Table S2). MSI-positive tumors also had higher rates of *BRAF* mutations (41.7% vs 3.8%, *P* < 0.001), *ATM* mutations (25% vs 3.8%, *P* = 0.029), *BLM* mutations (50% vs 1.3%, *P* < 0.001), *NOTCH1* mutations (16.7% vs 1.3%, *P* = 0.046), *CHEK2* mutations (16.7% vs 1.3%, *P* = 0.046) and *ROS1* mutations (16.7% vs 0.3%, *P* = 0.046, Table 1).

**Table 1:** Mutation profile of the most frequently altered genes in our study population by microsatellite instability status and tumor site.

	Microsatellite Instability					Tumor Site				
	MSI negative (N = 78)		MSI positive (N = 12)		*P* value	Proximal Colon (N = 20)		Distal Colon (N = 71)		*P* value
	n	(%)	n	(%)		n	(%)	n	(%)	
*APC*	59	(75.6)	5	(41.7)	0.034**	10	(50.0)	55	(77.5)	0.025*
*TP53*	51	(65.4)	1	(8.3)	<0.001*	4	(20.0)	48	(67.6)	<0.001*
*KRAS*	45	(57.7)	3	(25.0)	0.059*	3	(15.0)	45	(63.4)	<0.001*
*PIK3CA*	12	(15.4)	2	(16.7)	0.999**	4	(20.0)	10	(14.1)	0.499**
*FBXW7*	8	(10.3)	2	(16.7)	0.617**	2	(10.0)	8	(11.3)	0.999**
*NF1*	5	(6.4)	3	(25.0)	0.070**	3	(15.0)	5	(7.0)	0.367**
*TGFBR2*	0	(0.0)	8	(66.7)	<0.001**	6	(30.0)	2	(2.8)	0.001**
*ATM*	3	(3.8)	3	(25.0)	0.029**	4	(20.0)	2	(2.8)	0.020**
*BRAF*	3	(3.8)	5	(41.7)	0.001**	8	(40.0)	0	(0.0)	<0.001**
*BLM*	1	(1.3)	6	(50.0)	<0.001**	5	(25.0)	2	(2.8)	0.005**
*MSH6*	0	(0.0)	4	(33.3)	<0.001**	2	(10.0)	2	(2.8)	0.209**
*NOTCH1*	1	(1.3)	2	(16.7)	0.046**	1	(5.0)	2	(2.8)	0.530**
*NRAS*	4	(5.1)	0	(0.0)	0.999**	0	(0.0)	4	(5.6)	0.572**
*PTEN*	2	(2.6)	2	(16.7)	0.084**	3	(15.0)	1	(1.4)	0.032**
*CHEK2*	1	(1.3)	2	(16.7)	0.046**	1	(5.0)	2	(2.8)	0.530**
*ERBB3*	3	(3.8)	0	(0.0)	0.999**	0	(0.0)	3	(4.2)	0.999**
*ERBB4*	3	(3.8)	0	(0.0)	0.999**	1	(5.0)	2	(2.8)	0.530**
*NOTCH2*	0	(0.0)	3	(25.0)	0.002**	2	(10.0)	1	(1.4)	0.120**
*ROS1*	1	(1.3)	2	(16.7)	0.046**	2	(10.0)	1	(1.4)	0.120**
*STAT5B*	0	(0.0)	3	(25.0)	0.002**	2	(10.0)	1	(1.4)	0.120**

*Chi-squared test; **Fisher Exact Test; MSI: microsatellite instability.

In contrast, MSI-negative tumors showed a higher frequency of *APC* (75.6% vs 41.7%, *p* = 0.034) and *TP53* mutations (65.4% vs 8.3%, *p* < 0.001, Table 1), and some genes were exclusively altered in MSI-negative tumors, but without statistical significance (Supplementary Table S2).

### Differences between proximal and distal colon

Of the 91 tumor samples sequenced, 71 (78%) were from the distal colon, and 20 (22%) were from the proximal colon. The proximal colon had a higher frequency of MSI-positive tumors than the distal colon (50% versus 2.9%, *P* < 0.001). We found significant differences between the mutation profile of tumors from the proximal and distal colon (Fig. 1 and Table 1). Proximal colon tumors showed higher mutation frequencies than distal tumors on *TGFBR2* (30.0% vs 2.8%, *P* < 0.001), *ATM* (20.0% vs 2.8%, *P* = 0.020), *BLM* (25.0% vs 2.8%, *P* = 0.005), and *PTEN* (15.0% vs 1.4%, *P* = 0.032) genes. *BRAF* mutations were exclusively found in tumors from the proximal colon (40.0% vs 0.0%, *P* < 0.001), as well as mutations in the *EGFR*, *JAK1*, *MAP2K4*, *MAP3K1*, *NBN*, *ALK* genes (each of these mutated in 2 tumors), and *BRCA1*, *BRIP1*, *CREBBP*, *FLT3*, *FLT4*, *MLH1*, *MPL* and *NT5C2* genes (each of these mutated in 1 tumor).

In contrast, the distal colon showed a higher frequency than the proximal colon of *APC* (77.5% vs 50.0%, *P* = 0.025), *TP53* (67.6% vs 20.0%, *P* < 0.001) and *KRAS* (63.4% vs 15.0%, *P* < 0.001) gene mutations (Table 1). Mutations in *NRAS* (mutated in 4 tumors), *ERBB3* (mutated in 3 tumors), *RET*, *AKT1*, *AR*, *ARAF*, *BARD1*, *CDKN2A*, *GATA2*, *IDH2 MRE11A*, *NTRK1*, *PALB2* and *SMO* genes (each of these mutated in 1 tumor) were exclusively found in the distal colon.

### Mutation profile and genetic ancestry markers

We further assessed the ancestry of the patients by a panel of ancestry informative markers (AIMs) to estimate the proportion of ancestral background. The proportion of European genetic ancestry was predominant in our study population (median 83.1%, interquartile range 76.6% and 87.8%), followed by Native American (median 4.1%, interquartile range 2.9% and 7.2%), Asian (median 3.4%, interquartile range 2.8% and 5.1%) and African (median 3.2%, interquartile 2.1% and 7.9%) (Supplementary Fig. S4).

Patients with the highest proportion of African ancestry (those with African ancestry proportions higher than 7.9%) had more frequent *NF1* and *BRAF* mutations when compared with patients with intermediate and lowest proportions of African ancestry (*P* = 0.045 and 0.031, respectively, Supplementary Table S3). Patients with the highest proportion of Asian ancestry (those with a proportion of Asian ancestry higher than 5.1%) also had more *NF1* mutations than those patients with intermediate and low Asian ancestry (*P* = 0.037, Supplementary Table S3). Patients with the highest proportion of Native American ancestry (those with a proportion of Native American ancestry higher than 7.2%) had fewer *TP53* and *PIK3CA* mutations than those patients with the intermediate and the lowest proportion of Native American ancestry (*P* = 0.002 and 0.024, respectively, Supplementary Table S3).

## Discussion

In this study, we investigated mutations within 150 selected oncogenes and tumor suppressor genes in a Brazilian cohort. To the best of our knowledge, this is the largest mutation profile study in this population. Overall, our results showed that our population had a similar mutation profile to other populations (Table 2), with alterations in canonical driver genes, such *as APC*, *KRAS*, *TP53*, *PIK3CA*, and *FBXW7*. The differences between the proximal and distal colon were also highlighted in Brazilian patients, and the admixed features of our population were revealed by ancestry markers.

**Table 2:** Comparison of mutation frequency across different public data (cBioPortal) and studies.

Gene	Present Study (n = 91)		TCGA (n = 224)		DFCI (n = 619)		Genentech (n = 72)		ICGC: COCA-CN (321)		Salem *et al*. (2413)	
	N	(%)	N	(%)	N	(%)	N	(%)	N	(%)	N	(%)
*APC*	65	(71.4)	168	(75.0)	361	(58.3)	29	(40.3)	173	(53.9)	1513	(62.7)
*TP53*	51	(56.0)	121	(54.0)	320	(51.7)	40	(55.6)	158	(49.2)	1641	(68.0)
*KRAS*	48	(52.7)	94	(42.0)	173	(27.9)	37	(51.4)	118	(36.8)	1156	(47.9)
*PIK3CA*	14	(15.4)	45	(20.1)	132	(21.3)	22	(30.6)	60	(18.7)	326	(13.5)
*FBXW7*	10	(11.0)	37	(16.5)	86	(13.9)	15	(20.8)	59	(18.4)	—	—
*BRAF*	8	(8.8)	21	(9.4)	127	(20.5)	6	(8.3)	51	(15.9)	168	(7.0)
*NF1*	8	(8.8)	8	(3.6)	45	(7.3)	4	(5.6)	66	(20.6)	—	—
*TGFBR2*	8	(8.8)	23	(10.3)	29	(4.7)	2	(2.8)	25	(7.8)	—	—
*BLM*	7	(7.7)	10	(4.5)	12	(1.9)	0	(0.0)	25	(7.8)	—	—
*ATM*	6	(6.6)	25	(11.2)	64	(10.3)	13	(18.1)	53	(16.5)	34	(1.4)
*MSH6*	4	(4.4)	15	(6.7)	27	(4.4)	3	(4.2)	25	(7.8)	—	—
*NRAS*	4	(4.4)	20	(8.9)	27	(4.4)	2	(2.8)	17	(5.3)	—	—
*PTEN*	4	(4.4)	8	(3.6)	51	(8.2)	4	(5.6)	30	(9.3)	37	(1.5)
*ERBB*3	3	(3.3)	14	(6.3)	36	(5.8)	6	(8.3)	31	(9.7)	—	—
*ERBB4*	3	(3.3)	19	(8.5)	37	(6.0)	8	(11.1)	46	(14.3)	—	—
*NOTCH1*	3	(3.3)	0	(0.0)	65	(10.5)	6	(8.3)	38	(11.8)	—	—
*CHEK2*	3	(3.3)	1	(0.4)	36	(5.8)	5	(6.9)	22	(6.9)	—	—
*NOTCH2*	3	(3.3)	12	(5.4)	25	(4.0)	4	(5.6)	63	(19.6)	—	—
*ROS1*	3	(3.3)	13	(5.8)	31	(5.0)	2	(2.8)	46	(14.3)	—	—
*STAT5B*	*3*	(3.3)	3	(1.3)	15	(2.4)	3	(4.2)	12	(3.7)	—	—
*BRCA*2	2	(2.2)	10	(4.5)	42	(6.8)	5	(6.9)	35	(10.9)	—	—
*BRD*2	2	(2.2)	0	(0.0)	0	(0.0)	0	(0.0)	16	(5.0)	—	—
*EGFR*	2	(2.2)	10	(4.5)	28	(4.5)	4	(5.6)	53	(16.5)	—	—
*JAK1*	2	(2.2)	4	(1.8)	19	(3.1)	2	(2.8)	25	(7.8)	—	—
*MAP*2*K4*	2	(2.2)	11	(4.9)	13	(2.1)	2	(2.8)	16	(5.0)	—	—
*MAP3K1*	*2*	(2.2)	5	(2.2)	26	(4.2)	1	(1.4)	28	(8.7)	—	—
*MTOR*	*2*	(2.2)	17	(7.6)	50	(8.1)	8	(11.1)	42	(13.1)	—	—
*NBN*	*2*	(2.2)	2	(0.9)	20	(3.2)	1	(1.4)	26	(8.1)	—	—

TCGA: Comprehensive molecular characterization of human colon and rectal cancer^7^; DFCI: Genomic Correlates of Immune-Cell Infiltrates in Colorectal Carcinoma^15^; Genentech: Recurrent R-spondin fusions in colon cancer^19^; ICGC: COCA-CN: Colorectal cancer from non-Western samples (China)^30^; Salem *et al*.: Comparative molecular analyses of left-sided colon, right-sided colon, and rectal cancers^20^.

*APC* mutations are a common event in CRC, being an early and a general event for tumorigenesis progression^14^. Here, we showed that *APC* was the most frequently mutated gene, similar to several other studies demonstrated in different populations^7,15–17^ (Table 2). Additionally, as previously reported, we found that *APC* mutations were more frequent in distal colon and MSI-negative tumors^6,16^.

*TP53* is a ubiquitous driver gene in several cancer types^18^, and one of the most frequently altered genes in colorectal cancer, ranging from 51.7 to 68% of the cases^7,16,19,20^ (Table 2). Our study population showed a similar frequency of *TP53* gene alterations when compared with the TCGA frequency and studies in other populations^7,21^, showing the highest frequency in non-hypermutated tumors (MSI-negative tumors) and in distal colon tumors^7,17,22,23^.

The MAPK-ERK signaling pathway is significantly altered in CRCs and other tumors^18^. This pathway is constitutively activated by alterations in several driver genes, including *KRAS*, *BRAF*, and *NRAS*^7^. We observed that 68% of cases had a mutation in at least one gene in the MAPK-ERK pathway, in accordance with TCGA data (59.3%)^7,17^. Individually, we observed a high frequency of *KRAS* mutations (52.7%) that was slightly higher than the one reported in TCGA data (42%) and those found in other populations^15,17,21,24^ (Table 2). Moreover, previous studies on the prevalence of *KRAS* mutations carried out in Brazilian populations showed a lower frequency (31.9–49.2%) of mutations^25,26^. The higher frequency observed in the present study in comparison to those observed in other Brazilian studies may be associated with the higher sensitivity and coverage of the NGS approach used in our study and the high prevalence of early-stage and localized disease cases included in our study (78% stages 0, I and II). In agreement with other studies, most mutations in *the KRAS* gene were found at protein residues 12 and 13, with predominant Gly12Asp mutations^11,27^. Epidemiological and molecular observations showed that the proximal and distal colon exhibited distinct features. *KRAS* mutations are slightly higher in the proximal colon^20,28,29^. However, in our study, a predominance of *KRAS* mutations was observed in tumors from the distal colon. We also found a high mutation frequency in the *NF1* gene (Table 2), and interestingly, this frequency was associated with a high proportion of Asian ancestry, corroborating the known higher frequency of *NF1* mutations in the Asian population^30^.

*BRAF* mutations are reported in less than 10% of cases (Table 2) and have been related to MSI-positive and proximal colon tumors in several studies^6,7,17,20^. Here, we confirmed these observations and found a similar overall frequency of *BRAF* mutations, including the predominance of activating V600E type mutation^31^. Some non-V600E mutations, such as mutations at the D594 site, were shown to have either impaired or complete loss of kinase activity^32^, although constitutive activation of MAPK-ERK by these mutations has been shown to coincide with other mechanisms of activation, such as *NF1* deletions^33^. Non-V600E, such as the G469V mutation found here, has high kinase activity but acts in a different way than the V600E mutation^33^. The *NRAS* gene showed a lower frequency of mutation compared to the TCGA data, but it was similar to other large-scale studies (Table 2). Moreover, the significant pattern of mutually exclusivity of *KRAS*, *BRAF* and *NRAS* mutations found here has been observed in several studies^7,15,17,34^.

The PI3K pathway is another common pathway altered in CRC, leading to cell survival when activated^7,10,18^. Data from TCGA showed that genes from the PI3K signaling pathway were altered in approximately 25.9% of patients^7,17^, similar to the frequency reported in our study (23.1%). The *PIK3CA* gene seems to be the principal gene mutated in this pathway in CRCs^7,15^, showing activating mutations mainly on the PI3Ka domain (helicase), in addition to other already known activating mutations, such as H1047R and N345K/S found in the present study^7,35^. The *PTEN* gene is known to revert the action of PI3Ks^36^, and its inactivation is observed at a lower frequency in CRCs^7^ (1.5–9.3%, Table 2) by mutation^7,37^, as reported in our study (4.4%). The association between *PTEN* mutations and proximal colon localization has been observed previously^23^. Although *MTOR* was found to be mutated in CRCs with a range of 7.6–13.1% in large cohort studies (Table 2), these mutations are poorly characterized^38^. Mutations in *MTOR* site S2215, as we found here, seems to be the most frequently altered site across diverse cancer types and has been shown to increase the activity of mTOR protein^38^. The other *MTOR* (T2444M) mutation observed here has not been well characterized, but it affects the C-terminal region related to mTOR activation^38^. A low frequency of *AKT1* has been found across large cohort studies (Table 2), and the E17K mutation observed here has been related to the constitutive activation of *AKT1* protein^39^.

We found that 15.4% of tumors harboring missense mutations in members of the RTK family were lower than the frequency observed in TCGA data (22.3%)^7,17^. Although activating events in some RTK proteins can be observed by rearrangements in CRCs, such as *ROS1*^40^, *NTRK1*^40^, *ALK*^40^, and *RET*^41^, missense mutations in these genes are found across several studies (Table 2), but the oncogenic potential of such mutations is not completely understood in CRC. *RET* missense mutations promote tumor progression in thyroid cancer^42^ and are shown to reduce apoptosis in colorectal cancer cell lines^42^. Interestingly, *ALK*, *ROS1*, and *NTRK1* missense mutations have been observed in solid tumors resistant to tyrosine receptor kinase inhibitors^43–45^.

We found known activating mutations present in the protein kinase domain *EGFR* T725M^17,46^ and P848L^47^, as well as in *FLT3*^17,46^. Other mutations affecting the protein kinase domain of the *ROS1*, *ERBB4*, and *ALK* genes were also observed, but their role in oncogeneses is unknown^47^. Mutations that occur outside the protein kinase domain also play a role in the activation of RTKs, such as *ERBB3* mutations, which promote the growth of colonic cells^48^ and *ERBB4* mutations, which promote an increase in the kinase activity and transformation ability in melanoma cells^49^.

The Notch signaling pathway is important in maintaining the proliferative compartment of intestinal crypts^50^ and has been linked to tumor progression^51^. Here, we observed gene alterations in this pathway in 16.5% of the tumors, which was slightly lower when compared to the frequency observed on TCGA data for the same pathway (20.1%)^7,17^. *FBXW7* is one of the regulators of Notch protein^52,53^, and its loss seems to lead to the activation of this pathway^54^. The frequency of *FBXW7* mutations in our population was compared with those reported in other populations (Table 2)^55^. We observed mutations affecting hotspots of *FBXW7*, such as residues 465 and 505, that are important sites for substrate binding^56^, and frameshift mutations that lead to premature termination of the protein and have also been found affecting this gene^7,15,17^. The frequency of alterations in *NOTCH1* and *NOTCH2* genes is variable (Table 2), and missense activating mutations in both *NOTCH1* and *NOTCH2* have been observed in hematological tumors^57,58^. The pattern of mutual exclusivity between mutations in the *NOTC*H2 and *FBXW7* genes that we found here has not been observed in large cohort studies^7,15,17^.

The presence of MSI was found in 13.3% of cases, in accordance with previous studies^4,6,59^. In addition, MSI was associated with tumors in the proximal colon and with those exhibiting a higher mutation burden^6,7,19^. As previously reported, we observed an association between *ATM*^7,17^, *BLM*^7,17^, and *TGFRB2*^6,7,17^ mutations and MSI and the proximal colon.

Epidemiological evidence suggests that ethnicity has a high impact on cancer incidence and mortality^60^, and differences in the prevalence of cancer driver mutations have been observed in several cancer types, including CRC^61^. Our population is known to be diverse^62^, and our results are in agreement with these observations, showing differences in the proportions of each of the main four population origins compared with previous studies^62,63^. Our patients with a higher African ancestry proportion had a higher frequency of *BRAF* mutations, contrasting with a previous study by Staudacher *et al*.^64^. We did not observe an association between the frequency of *KRAS* mutations and the African ancestry that was reported previously^64^. These disparities may reside in the fact that, although we have an admixture population study, we have a low frequency of self-declared African ancestry and a low frequency of proportion of African ancestry than observed in other population studies^62,63^. In contrast, the association of *NF1* mutations and African ancestry has been observed previously^65^. Moreover, the observation that *NF1* mutations were associated with the high Asian ancestry proportion was consistent with the data from ICGC^30^. Although ethnicity association with *PIK3CA* mutations has been observed in different populations^65,66^, this is the first study to demonstrate an inverse correlation between *PIK3CA* mutations and a high Native American ancestry background.

Despite the major findings, one limitation of the present study lies in the preselection of a panel of known cancer genes, and known genes related to CCR carcinogenesis were not included in the analysis, such as *SMAD4* (altered in 11.6–12.9% of CRC), *TTN* (35.7–48.1%) and *SYNE1* (21.0–48.1%)^7,15,17^. Moreover, using whole-genome or exome approaches, other putative CCR cancer-related genes could have been identified in the Brazilian context.

In conclusion, our study constitutes the largest mutation landscape of Brazilian colorectal cancer patients. This study paves the way for a better comprehension of the major alterations identified and could guide better-tailored therapy for colorectal cancer in the Brazilian population.

## Materials and Methods

### Tissue samples

Ninety-one patients diagnosed with colorectal adenocarcinomas and admitted for a surgical procedure at Barretos Cancer Hospital, Barretos, SP, Brazil, were evaluated. The main clinicopathological features are summarized in Table 3. The mean age of patients was 61 years old, and the majority of cases had early-stage and localized disease (0, I and II).

**Table 3:** Characteristics of the study population.

Characteristics (N = 91)	Frequency	(%)
	Mean	(range)
Age at diagnostic	61.18	(29–88)
Gender		
Male	49	53.8
Female	42	46.2
Self-Assessed Skin Color (n = 89)		
White	76	85.4
Black	2	2.2
Brown	10	11.2
Yellow	1	1.1
Primary disease site		
Right-sided	20	22.0
Cecum	4	20.0
Ascending colon	9	45.0
Transverse colon	7	35.0
Left-sided	71	78.0
Sigmoid colon	31	43.7
Rectosigmoid junction	15	21.1
Rectum	25	35.2
Stage (at diagnosis)		
0	3	3.3
I	30	33.0
II	38	41.8
III	16	17.6
IV	4	4.4
TNM		
Primary Tumor (T)		
Tis	5	5.5
T1	6	6.6
T2	28	30.8
T3	42	46.2
T4 (a,b)	10	11.0
Regional Lympho Nodes (N)		
N0	69	75.8
N1 (a,b,c)	15	16.5
N2 (a,b)	7	7.7
Distant Metastasis (M)		
M0	87	95.6
M1a	3	3.3
M1b	1	1.1
Histologic Grade		
I	10	11.0
II	77	84.6
III	4	4.4
Angiolymphatic invasion (n = 90)	17	18.9
Perineural invasion (n = 86)	6	7.0
Adjuvant chemotherapy	30	33.0
Radiotherapy (n = 90)	4	4.4
Tumor Recurrence (n = 89)	3	3.4
MSI (n = 90)		
MSI-negative	78	86.7
MSI-positive	12	13.3

All included patients signed an informed consent form. Both tumors and blood were obtained and immediately processed and stored at −80 °C at Barretos Cancer Hospital Biobank. The present study was approved by the Barretos Cancer Hospital Institutional Review Board (Project n° 1060/2015, protocol CAAE: 51770115.6.0000.5437). All methods were performed in accordance with the relevant guidelines and regulations.

### DNA isolation

Tumor DNA was isolated from fresh-frozen tissue using the QIAsymphony DNA Mini Kit following the Tissue_200 protocol for automated isolation in the QIAsymphony (QIAGEN, Hilden, Germany). DNA from leucocytes of peripheral blood was isolated using the QIAmp DNA Blood Mini Kit (QIAGEN, Hilden, Germany) following the manufacturer’s instructions. DNA quantity and quality were assessed by Qubit (Thermo Fisher Scientific, Waltham, MA, USA).

### Microsatellite instability analyses

The MSI status was evaluated using a multiplex PCR comprising six quasi-monomorphic mononucleotide repeat markers (BAT-25, BAT-26, NR-21, NR-24, NR-27 and HSP110) as described previously^59^. The MSI analyses were performed using GeneMapper v4.1 software (Applied Biosystems), and the status was classified as stable (MSS) when none of the markers were unstable. The status was MSI-Low (MSI-L) when one of the markers was unstable and MSI-positive when two or more of the markers were unstable. MSS and MSI-L were considered MSI-negative^59^.

### Mutation profile

The mutation profile of a commercial panel of 150 cancer-related genes (for a list of genes, see Supplementary Information) was conducted at the Mendelics Genetics company (São Paulo, SP, Brazil, (https://www.mendelics.com/oncologia/). The panel analyzed all coding regions of the 150 cancer-related genes. For sequencing, paired tumor and blood DNA libraries were prepared using the Nextera Rapid Capture Custom Enrichment Kit (Illumina, San Diego, CA, USA). Libraries were quantified using a Qubit fluorometer (Thermo Fisher Scientific, Waltham, MA, USA), and their quality was evaluated using an Agilent 2100 Bioanalyzer (Agilent Technologies, Santa Clara, CA, USA). Cluster generation and sequencing were performed on the Illumina HiSeq. 4000 following the manufacturer’s instructions. Paired-end reads from Illumina sequencing were processed by script bcl2fastq (v. 2.17.1.14) and aligned against the human genome reference build GRCh37 using Burrows-Wheeler Aligner (BWA, version 0.7.13)^67^. The somatic variants were called by a VarScan2 algorithm^68^. The variants with artifacts due to indel reads at their position or less than 10% or more than 90% of variant supporting reads on one strand were removed. The variants were further filtered to remove those with fewer than 10 reads covering the variant and with less than 5% variant allele frequency. The annotation of variants was performed using Ensembl Variant Effect Predictor (VEP)^69^.

To identify driver mutations on tumors, we used the Cancer Genome Interpreter – CGI^70^. After the classification of mutations by the CGI, we excluded mutations that were not classified as cancer driver mutations or not predicted as a driver by the OncoMut algorithm that CGI used. Therefore, mutations identified as polymorphisms (high allele frequency) or those predicted as neutral or passenger for oncogenesis, and those found in DNA sequences outside coding regions were excluded.

We further validated mutations on codons 12/13 and codon 61 of the *KRAS* gene using the cobas^®^ KRAS Mutation Test (F. Hoffmann-La Roche, Basel, Switzerland) following the manufacturer’s recommendations. We also performed PCR-followed by direct Sanger sequencing for a subset of samples to confirm mutations in the *APC* and *TP53* genes. The PCR and Sanger sequencing conditions were previously reported by our group^71,72^

### Genetic ancestry markers

The genetic ancestry of patients was determined by 46 autosomal ancestry informative markers (AIMs), consisting of insertion and deletion polymorphisms and using the genetic data of the Human Genome Diversity Project – center d’Etude du Polymorphisme Humain (HGDP-CEPH), according to Pereira *et al*.^63^. We classified the proportion of ancestry by tertiles as high, intermediary and low for each of the main populations: African, European, Asian and Native American^73^.

### Statistical analyses

Chi-square and Fisher’s exact tests were used to set differences between mutated and nonmutated genes and patient characteristics. Mutation types and mutation burden differences between MSI-positive and MSI-negative tumors were determined by chi-square and Mann-Whitney tests, respectively. All statistical analyses were performed using SPSS software (v.20) and R 3.5.0 software. Mutation mapper figures were generated using cBioPortal. Oncoplots and ancestry charts were generated using GenVisR and ggplot2 packages (versions 1.14 and 3.1.0, respectively) in R 3.5.0 software.

## Supplementary information

Supplementary Material

## Data Availability

Data that support the findings are available from Dr. Denise Peixoto Guimarães and Dr. Rui Manual Reis and are not publicly available due patient personal information. However, these data are available upon reasonable request and with permission of the Dr. Rui Manuel Reis (Scientific and Executive Director of the Molecular Oncology Research Center, Barretos Cancer Hospital).
