# Supplementary material for: Mutation profiling of cancer drivers in Brazilian colorectal cancer
Source: Sci Rep. 2019 Sep 23;9:13687. doi: 10.1038/s41598-019-49611-1 (PMC6757044; doi:10.1038/s41598-019-49611-1)
Supplement: Supplementary file 1 — Supplementary Material [file 41598_2019_49611_MOESM1_ESM.pdf]

Mutation profiling of cancer drivers in Brazilian colorectal cancer

Authors: Wellington dos Santos; Thais Sobanski; Ana Carolina de Carvalho; Adriane Feijó Evangelista; Marcus Matsushita; Gustavo Berardinelli; Marco Antonio de Oliveira; Rui Manuel Reis; Denise Peixoto Guimarães.

Supplementary Information

The panel of cancer-related genes sequenced include all coding sequences of the follow genes: *ABL1, AKT1, AKT2, AKT3, ALK, APC, AR, ARAF, ATM, AURKA, AURKB, AXIN1, AXL, BAP1, BARD1, BCL2, BCL2L1, BCL2L2, BLM, BRAF, BRCA1, BRCA2, BRD2, BRD3, BRD4, BRDT, BRIP1, BTK, CBFB, CCND1, CCND2, CCND3, CCNE1, CD22, CD274, CD79A, CD79B, CDH1, CDK12, CDK4, CDKN1A, CDKN2A, CHEK1, CHEK2, CREBBP, CRKL, DDR2, DDX3X, EGFR, EPHA7, ERBB2, ERBB3, ERBB4, ESR1, EZH2, FAM175A, FAS, FBXW7, FGFR1, FGFR2, FGFR3, FGFR4, FHIT, FLT1, FLT3, FLT4, FRS2, GATA2, GNA11, GNAQ, HDAC1, HDAC4, HDAC7, HGF, HRAS, IDH1, IDH2, IGF1R, JAK1, JAK2, JAK3, KDR, KIT, KRAS, MAP2K1, MAP2K2, MAP2K4, MAP3K1, MAPK1, MCL1, MDM2, MET, MLH1, MPL, MRE11A, MS4A1, MSH2, MSH6, MTOR, MUTYH, MYC, MYD88, NBN, NF1, NF2, NFE2L2, NFKBIA, NOTCH1, NOTCH2, NOTCH3, NRAS, NT5C2, NTRK1, PALB2, PDGFRA, PDGFRB, PDK1, PIK3CA, PIK3CB, PIK3CG, PMS2, PTCH1, PTEN, RAC1, RAD51, RAF1, RANBP2, RARA, RB1, RET, RICTOR, ROS1, RRM1, RUNX1, SDHB, SMO, SOX2, SRC, STAT3, STAT5B, STK11, TERC, TERT, TGFBR2, TP53, TSC1, TSC2, VEGFA, WT1, e XPO1*.

Table S1. List of alterations found on NGS, excluding silent alteration

Gene (number of mutations)	N (%)	Mutation Status (CGI)	Annotation on COSMIC or dbSNP
APC (95)	66 (72.5)		
p.Thr1556AsnfsTer3	5 (5.5)	Driver	COSM19695
p.Arg1114Ter	3 (3.3)	Driver	rs121913331,COSM13125
p.Arg232Ter	3 (3.3)	Driver	rs397515734,COSM13130
p.Gln1291Ter	3 (3.3)	Driver	COSM19072
p.Gln1338Ter	3 (3.3)	Driver	rs121913327,COSM13129
p.Val1822Asp	3 (3.3)	Polymorphism	rs459552,COSM3760871
p.Arg283Ter	2 (2.2)	Driver	COSM19679
p.Arg302Ter	2 (2.2)	Driver	rs137854568,COSM13862
p.Arg554Ter	2 (2.2)	Driver	rs137854573,COSM19040
p.Arg876Ter	2 (2.2)	Driver	rs121913333,COSM18852
p.Gln1406Ter	2 (2.2)	Driver	rs587782518,COSM19087
p.Gln1429Ter	2 (2.2)	Driver	rs74535574,COSM18836
p.Glu1317Ter	2 (2.2)	Driver	COSM19253
p.Glu1554Ter	2 (2.2)	Driver	COSM33818
p.Ala1299LeufsTer6	1 (1.1)	Driver	NA
p.Ala199CysfsTer53	1 (1.1)	Driver	NA
p.Ala731Pro	1 (1.1)	Driver	NA
p.Arg1171Cys	1 (1.1)	Passenger	rs201830995,COSM1580515
p.Arg1450Ter	1 (1.1)	Driver	rs121913332,COSM13127
p.Arg213Ter	1 (1.1)	Driver	rs587781392,COSM13134
p.Arg216Gln	1 (1.1)	Passenger	rs76685252,COSM3826545
p.Arg216Ter	1 (1.1)	Driver	rs62619935,COSM98420
p.Arg498Ter	1 (1.1)	Driver	NA
p.Arg499Ter	1 (1.1)	Driver	rs137854580,COSM29364
p.Arg805Ter	1 (1.1)	Driver	rs587779783,COSM19058
p.Asn1535MetfsTer30	1 (1.1)	Driver	NA
p.Asn862LysfsTer50	1 (1.1)	Driver	NA
p.Asp1058MetfsTer3	1 (1.1)	Driver	NA
p.Asp1569GlufsTer2	1 (1.1)	Driver	NA
p.Asp170ValfsTer4	1 (1.1)	Driver	COSM1432145

Gene (number of mutations)	N (%)	Mutation Status (CGI)	Annotation on COSMIC or dbSNP
p.Asp1860Tyr	1 (1.1)	Driver	NA
p.Cys1410Ter	1 (1.1)	Driver	COSM19592
p.Gln1294AspfsTer8	1 (1.1)	Driver	NA
p.Gln1367Ter	1 (1.1)	Driver	rs121913328,COSM13121
p.Gln1477ArgfsTer30	1 (1.1)	Driver	NA
p.Gln1529Ter	1 (1.1)	Driver	COSM1183187
p.Glu1209Ter	1 (1.1)	Driver	COSM167689
p.Glu1295Ter	1 (1.1)	Driver	COSM18961
p.Glu1306Ter	1 (1.1)	Driver	rs121913462,COSM18760
p.Glu1309AspfsTer4	1 (1.1)	Driver	rs763847228,COSM18764
p.Glu1322Ter	1 (1.1)	Driver	COSM18702
p.Glu1345Ter	1 (1.1)	Driver	COSM18759
p.Glu1374Ter	1 (1.1)	Driver	COSM19085
p.Glu1379Ter	1 (1.1)	Driver	rs121913326,COSM18834
p.Glu1408Ter	1 (1.1)	Driver	COSM18822
p.Glu1494LysfsTer19	1 (1.1)	Driver	COSM19349
p.Glu403LysfsTer51	1 (1.1)	Driver	NA
p.Glu425Ter	1 (1.1)	Driver	NA
p.Glu941Ter	1 (1.1)	Driver	COSM18853
p.Gly1120Arg	1 (1.1)	Passenger	NA
p.Gly1288Ter	1 (1.1)	Driver	COSM19577
p.Gly2250AlafsTer29	1 (1.1)	Driver	rs773874693
p.His1490TyrfsTer19	1 (1.1)	Driver	NA
p.Leu1129Ser	1 (1.1)	Passenger	rs143638171,COSM30751
p.Leu1488PhefsTer26	1 (1.1)	Driver	NA
p.Leu1488TyrfsTer19	1 (1.1)	Driver	COSM18999
p.Leu1972GlnfsTer2	1 (1.1)	Driver	NA
p.Leu2168Ile	1 (1.1)	Passenger	COSM1059629
p.Leu356Pro	1 (1.1)	Driver	NA
p.Leu779PhefsTer2	1 (1.1)	Driver	NA
p.Lys1437Ter	1 (1.1)	Driver	COSM18818
p.Lys311Asn	1 (1.1)	Driver	NA

Gene (number of mutations)	N (%)	Mutation Status (CGI)	Annotation on COSMIC or dbSNP
p.Lys756AsnfsTer5	1 (1.1)	Driver	NA
p.Phe1491LeufsTer16	1 (1.1)	Driver	COSM19623
p.Ser1205AlafsTer60	1 (1.1)	Driver	NA
p.Ser1315Ter	1 (1.1)	Driver	COSM19582
p.Ser1436ValfsTer37	1 (1.1)	Driver	COSM19607
p.Ser943Ter	1 (1.1)	Driver	COSM98439
p.Thr1556LeufsTer9	1 (1.1)	Driver	COSM19000
p.Trp685Ter	1 (1.1)	Driver	COSM1432191
p.Val1320GlyfsTer95	1 (1.1)	Driver	NA
p.Val1320Ter	1 (1.1)	Driver	COSM23635
p.Val1528Gly	1 (1.1)	Passenger	NA
TP53 (62)	52 (57.1)		
p.Gly245Ser	5 (5.5)	Driver	rs28934575,COSM6932,COSM121035,COSM121036,COSM3356965,COSM1640833,COSM121037
p.Pro72Arg	5 (5.5)	Polymorphism	rs1042522,COSM250061,COSM3766190,COSM3766193,COSM3766192,COSM3766191
p.Arg248Trp	4 (4.4)	Driver	rs121912651,COSM10656,COSM120007,COSM120005,COSM3388183,COSM1640831,COSM120006
p.Arg273His	4 (4.4)	Driver	rs28934576,TP53_g.13798G>A,COSM10660,COSM99729,COSM3356963,COSM1645335
p.Arg175His	3 (3.3)	Driver	rs28934578,COSM10648,COSM99914,COSM99022,COSM99023,COSM3355994,COSM1640851,COSM99024
p.Arg213Ter	3 (3.3)	Driver	rs397516436,COSM10654,COSM99618,COSM99615,COSM99616,COSM3378350,COSM1638393,COSM99617
p.Arg273Cys	2 (2.2)	Driver	rs121913343,COSM10659,COSM99933,COSM3355991,COSM1645518
p.Ile255del	2 (2.2)	Driver	COSM1480061,COSM1480062,COSM1480063
c.672+1G>T_p.X224_splice	1 (1.1)	Driver	COSM13586,COSM118941,COSM118939,COSM3723947,COSM1638407,COSM118940
p.Ala161Ser	1 (1.1)	Driver	COSM43549,COSM1610856,COSM1610857,COSM1610859,COSM3717664,COSM3717663,COSM1610858
p.Arg181His	1 (1.1)	Driver	rs397514495,COSM10738,COSM1386797,COSM1386798,COSM1386800,COSM3388202,COSM3388201,COSM1386799
p.Arg196Ter	1 (1.1)	Driver	rs397516435,COSM10705,COSM99668,COSM99665,COSM99666,COSM3378446,COSM1640847,COSM99667
p.Arg248Gln	1 (1.1)	Driver	rs11540652,COSM10662,COSM99602,COSM99020,COSM3356964,COSM1640830,COSM99021
p.Arg282Trp	1 (1.1)	Driver	rs28934574,COSM10704,COSM99925,COSM3378339,COSM1636702
p.Cys229ValfsTer18	1 (1.1)	Driver	COSM44323
p.Cys242Arg	1 (1.1)	Driver	COSM11738,COSM1386619,COSM1386620,COSM1386621
p.Cys242Phe	1 (1.1)	Driver	COSM10810,COSM129834,COSM129835,COSM3378347,COSM1646852,COSM129836
p.Gln100Ter	1 (1.1)	Driver	COSM44032,COSM1189391,COSM1189392
p.Gln144Ter	1 (1.1)	Driver	rs757274881,COSM11245,COSM318146,COSM318145,COSM318147,COSM3820722,COSM2744941,COSM318148
p.Gln165Ter	1 (1.1)	Driver	rs730882001,COSM43632,COSM1172488,COSM1172489,COSM1172491,COSM3388213,COSM3388212,COSM1172490

Gene (number of mutations)	N (%)	Mutation Status (CGI)	Annotation on COSMIC or dbSNP
p.Glu180Lys	1 (1.1)	Driver	COSM43772,COSM1522501,COSM1522502,COSM1522504,COSM1522503
p.Gly187Ser	1 (1.1)	Driver	rs776167460,COSM44026,COSM437537,COSM437538,COSM437540,COSM3522697,COSM1636742,COSM437539
p.Gly266Arg	1 (1.1)	Driver	COSM10794,COSM1559475,COSM3388175,COSM1645293
p.Gly266Glu	1 (1.1)	Driver	rs193920774,COSM10867,COSM216410,COSM3388174,COSM1679491
p.His168ProfsTer13	1 (1.1)	Driver	NA
p.His193Arg	1 (1.1)	Driver	COSM10742,COSM308307,COSM308306,COSM308308,COSM3820719,COSM1740322,COSM308309
p.His193Asp	1 (1.1)	Driver	COSM44002,COSM251417,COSM251416,COSM251418,COSM251419
p.His193Leu	1 (1.1)	Driver	COSM11066,COSM99919,COSM99916,COSM99917,COSM3732881,COSM2744772,COSM99918
p.Ile195Asn	1 (1.1)	Driver	COSM44877,COSM1738250,COSM1738252,COSM1738254,COSM3403267,COSM1738251,COSM1738253
p.Ile195Phe	1 (1.1)	Driver	COSM44633,COSM129840,COSM129841,COSM129843,COSM3388199,COSM1731912,COSM129842
p.Ile255dup	1 (1.1)	Driver	NA
p.Lys132Arg	1 (1.1)	Driver	COSM11582,COSM308311,COSM308310,COSM3388223,COSM1646844,COSM308312
p.Met160AsnfsTer21	1 (1.1)	Driver	NA
p.Met160Ile	1 (1.1)	Driver	rs772354334,COSM43891
p.Phe109Ser	1 (1.1)	Driver	COSM45169,COSM148181,COSM148180
p.Pro152ArgfsTer18	1 (1.1)	Driver	COSM43792,COSM1180849,COSM1180850,COSM1180852,COSM1180851
p.Pro85Arg	1 (1.1)	Passenger	NA
p.Ser241Phe	1 (1.1)	Driver	rs28934573,COSM10812,COSM214171,COSM214170,COSM3522695,COSM1649402,COSM214172
p.Ser90LeufsTer59	1 (1.1)	Driver	COSM131024,COSM131025,COSM131026
p.Thr125Met	1 (1.1)	Driver	COSM44988,COSM168303,COSM168302,COSM3717682,COSM1750374
p.Tyr205His	1 (1.1)	Driver	COSM43642,COSM1159830,COSM1159831,COSM1159833,COSM1159832
p.Tyr236Cys	1 (1.1)	Driver	COSM10731,COSM116674,COSM116672,COSM3773303,COSM1646851,COSM116673
KRAS (49)	49 (53.8)		
p.Gly12Asp	16 (17.6)	Driver	rs121913529,COSM521,COSM1135366
p.Gly12Val	11 (12.1)	Driver	rs121913529,COSM520,COSM1140133
p.Gly13Asp	7 (7.7)	Driver	rs112445441,COSM532,COSM1140132
p.Gly12Ala	3 (3.3)	Driver	rs121913529,COSM522,COSM1140134
p.Gly12Cys	3 (3.3)	Driver	rs121913530,COSM516,COSM1140136
p.Ala146Thr	2 (2.2)	Driver	rs121913527,COSM19404,COSM1165198
p.Gln61His	2 (2.2)	Driver	rs17851045,COSM1135364,COSM1146992,COSM554,COSM555
c.*73T>C_3'UTR	1 (1.1)	Polymorphism	rs1137282,COSM3753105
p.Ala146Pro	1 (1.1)	Driver	COSM19905,COSM1140130

Gene (number of mutations)	N (%)	Mutation Status (CGI)	Annotation on COSMIC or dbSNP
p.Ala146Val	1 (1.1)	Driver	COSM19900,COSM1360827
p.Gly13Glu	1 (1.1)	Driver	COSM87280
p.Lys117Asn	1 (1.1)	Driver	COSM28519,COSM1562192
FLT4 (18)	12 (13.2)		
p.His890Gln	5 (5.5)	Driver	rs448012,COSM1486680,COSM449470
p.Arg1324Leu	3 (3.3)	Polymorphism	rs307821,COSM3761168
p.Thr494Ala	3 (3.3)	Polymorphism	rs307826,COSM3761172,COSM3761171,COSM3761173
p.Arg1146His	2 (2.2)	Polymorphism	rs1130379,COSM4159821,COSM4159820
p.Asn149Asp	2 (2.2)	Polymorphism	rs34221241,COSM4159823,COSM4159822
p.Arg119His	1 (1.1)	Passenger	rs751885535,COSM1471828,COSM1471827
p.Arg1320Trp	1 (1.1)	Passenger	rs150279372
p.Arg283Cys	1 (1.1)	Driver	rs368167894,COSM3854398,COSM3854397,COSM3854399
PIK3CA (17)	17 (18.7)		
p.Glu545Lys	4 (4.4)	Driver	rs104886003,COSM763,COSM125370
c.*5720delA_3'UTR	2 (2.2)	Polymorphism	NA
p.Glu542Lys	2 (2.2)	Driver	rs121913273,COSM760,COSM125369
p.Asn345Lys	1 (1.1)	Driver	rs121913284,COSM754,COSM132748
p.Asn345Ser	1 (1.1)	Driver	COSM13476,COSM1420790
p.Gln546Lys	1 (1.1)	Driver	rs121913286,COSM766,COSM255876
p.Glu545Ala	1 (1.1)	Driver	rs121913274,COSM12458,COSM297145
p.Glu545Gln	1 (1.1)	Driver	COSM27133,COSM295672
p.Glu81Lys	1 (1.1)	Driver	COSM27502,COSM271871
p.Gly118Asp	1 (1.1)	Driver	COSM751,COSM246588
p.His1047Arg	1 (1.1)	Driver	rs121913279,COSM775,COSM94986
p.Leu339Ile	1 (1.1)	Passenger	NA
FGFR4 (12)	9 (9.9)		
p.Gly388Arg	4 (4.4)	Polymorphism	rs351855,COSM1567768
p.Pro136Leu	4 (4.4)	Polymorphism	rs376618
p.Val10Ile	3 (3.3)	Polymorphism	rs1966265
p.Pro568GlnfsTer53	1 (1.1)	Passenger	NA
FBXW7 (11)	11 (12.1)		
p.Arg465Cys	1 (1.1)	Driver	COSM22932,COSM170727,COSM1154293,COSM170726,COSM170725

Gene (number of mutations)	N (%)	Mutation Status (CGI)	Annotation on COSMIC or dbSNP
p.Arg465His	1 (1.1)	Driver	COSM22965,COSM117310,COSM1149856,COSM117309,COSM117308
p.Arg465Leu	1 (1.1)	Driver	COSM33762,COSM673925,COSM673924,COSM673923
p.Arg505Cys	1 (1.1)	Driver	rs149680468,COSM22975,COSM108572,COSM1154290,COSM108571,COSM74637
p.Arg505His	1 (1.1)	Driver	COSM133117,COSM25812,COSM287374,COSM3127975,COSM287373,COSM287372
p.Glu287Asp	1 (1.1)	Passenger	NA
p.Leu288Phe	1 (1.1)	Driver	NA
p.Leu594Pro	1 (1.1)	Driver	COSM1427660,COSM1427661,COSM1427663,COSM1427664,COSM1427662
p.Met268AspfsTer18	1 (1.1)	Driver	COSM1052121,COSM1052122,COSM1594349,COSM1052124,COSM1052123
p.Ser668ValfsTer39	1 (1.1)	Driver	COSM34018,COSM1427622,COSM1427624,COSM1427625,COSM1427623
p.Tyr545Cys	1 (1.1)	Driver	COSM22989,COSM1052084,COSM1154289,COSM1052086,COSM1052085
NF1 (11)	9 (9.9)		
p.Ala2032Val	1 (1.1)	Driver	NA
p.Ala548Asp	1 (1.1)	Driver	NA
p.Arg1462Trp	1 (1.1)	Driver	COSM472532,COSM472531
p.Arg2450Ter	1 (1.1)	Driver	COSM24487,COSM303898
p.Arg2832His	1 (1.1)	Driver	rs587781523
p.Arg652His	1 (1.1)	Passenger	rs587778549
p.Gln1360SerfsTer20	1 (1.1)	Driver	NA
p.Lys2372Asn	1 (1.1)	Driver	NA
p.Phe1536Ser	1 (1.1)	Driver	NA
p.Ser1838TyrfsTer23	1 (1.1)	Driver	NA
p.Tyr628ThrfsTer3	1 (1.1)	Driver	NA
ATM (10)	8 (8.8)		
p.Lys2811SerfsTer46	2 (2.2)	Driver	rs759472682,COSM1351020,COSM1351019
p.Arg2832His	1 (1.1)	Driver	rs529296539
p.Arg3008His	1 (1.1)	Driver	rs587781894,COSM21626,COSM1139600
p.Asp814Glu	1 (1.1)	Passenger	rs3218695
p.Gln445Ter	1 (1.1)	Driver	NA
p.Glu376Ter	1 (1.1)	Driver	NA
p.Gly2024Trp	1 (1.1)	Driver	NA
p.His1213Asn	1 (1.1)	Driver	NA
p.Leu1561Ile	1 (1.1)	Passenger	NA

Gene (number of mutations)	N (%)	Mutation Status (CGI)	Annotation on COSMIC or dbSNP
BLM (10)	8 (8.8)		
p.Asn515MetfsTer16	5 (5.5)	Driver	rs772495493,COSM252959
p.Asp757ThrfsTer4	1 (1.1)	Driver	rs747341586,COSM1375443
p.Ile1150Val	1 (1.1)	Passenger	NA
p.Pro707Ser	1 (1.1)	Passenger	rs146077918,COSM3505421
p.Pro868Leu	1 (1.1)	Driver	rs2227935
p.Val1321Ile	1 (1.1)	Polymorphism	rs7167216
BRAF (10)	9 (9.9)		
p.Val600Glu	6 (6.6)	Driver	rs113488022,COSM476
p.Ala404CysfsTer9	1 (1.1)	Passenger	rs777474487,COSM1448631
p.Arg239Ter	1 (1.1)	Passenger	NA
p.Asp594Glu	1 (1.1)	Driver	COSM253330
p.Gly469Val	1 (1.1)	Driver	rs121913355,COSM459
BRCA1 (10)	5 (5.5)		
p.Lys1183Arg	2 (2.2)	Polymorphism	rs16942,COSM148277,COSM3755561
p.Pro871Leu	2 (2.2)	Polymorphism	rs799917,COSM148278,COSM3755564
p.Ser1634Gly	2 (2.2)	Neutral	rs1799966,COSM3755560,COSM3755559
p.Asn1509IlefsTer17	1 (1.1)	Driver	NA
p.Glu1038Gly	1 (1.1)	Polymorphism	rs16941,COSM3755563,COSM3755562
p.Ser1040Asn	1 (1.1)	Polymorphism	rs4986852,COSM1166811
p.Thr1196Lys	1 (1.1)	Passenger	rs80356944
MAP3K1 (10)	7 (7.7)		
p.Asp806Asn	2 (2.2)	Polymorphism	rs702689,COSM4003565,COSM4003564
p.Ser939Cys	2 (2.2)	Polymorphism	rs45556841
p.Thr949del	2 (2.2)	Polymorphism	rs5868032
p.Val906Ile	2 (2.2)	Polymorphism	rs832582
p.Ala1373Val	1 (1.1)	Driver	NA
p.Ala618SerfsTer28	1 (1.1)	Driver	NA
TGFBR2 (9)	8 (8.8)		
p.Lys153SerfsTer35	6 (6.6)	Driver	COSM1180952,COSM1744957
p.Lys153AlafsTer3	2 (2.2)	Driver	COSM1645197,COSM1645196
p.Arg218Trp	1 (1.1)	Driver	rs61762550

Gene (number of mutations)	N (%)	Mutation Status (CGI)	Annotation on COSMIC or dbSNP
RANBP2 (8)	6 (6.6)		
p.Arg2076Gln	1 (1.1)	Passenger	NA
p.Asn1036Tyr	1 (1.1)	Passenger	NA
p.Asp1926Asn	1 (1.1)	Passenger	NA
p.Cys2737LeufsTer5	1 (1.1)	Passenger	NA
p.Cys2737ValfsTer35	1 (1.1)	Passenger	COSM1398708,COSM1398707
p.Cys707Arg	1 (1.1)	Passenger	NA
p.Glu1213Ter	1 (1.1)	Passenger	rs767158817
p.Thr1004LeufsTer12	1 (1.1)	Passenger	NA
ALK (7)	7 (7.7)		
p.Ala421Ser	1 (1.1)	Passenger	NA
p.Arg1120Trp	1 (1.1)	Driver	rs774951734
p.Asp1529Glu	1 (1.1)	Polymorphism	rs1881421,COSM3758201
p.Pro1620Thr	1 (1.1)	Passenger	NA
p.Thr533Ile	1 (1.1)	Passenger	NA
p.Val351Ala	1 (1.1)	Passenger	NA
p.Val476Ala	1 (1.1)	Polymorphism	rs35093491,COSM3758202
BARD1 (7)	5 (5.5)		
p.Arg378Ser	4 (4.4)	Polymorphism	rs2229571,COSM3757931,COSM3757932
p.Pro24Ser	1 (1.1)	Polymorphism	rs1048108,COSM442275,COSM1482743
p.Ser192LeufsTer20	1 (1.1)	Driver	NA
p.Val507Met	1 (1.1)	Polymorphism	rs2070094,COSM3757929,COSM3757930
NOTCH1 (7)	5 (5.5)		
p.Arg1356Cys	1 (1.1)	Passenger	rs587778567
p.Arg2070Gln	1 (1.1)	Driver	rs751048658,COSM1259321,COSM1259320
p.Gln2394Ter	1 (1.1)	Driver	COSM13062
p.Ile1981Thr	1 (1.1)	Driver	NA
p.Pro2222Leu	1 (1.1)	Driver	rs779125725
p.Thr1379Met	1 (1.1)	Passenger	NA
p.Val2476Met	1 (1.1)	Passenger	rs763785541
BRDT (6)	5 (5.5)		
p.Gln62Lys	2 (2.2)	Polymorphism	rs10783071

Gene (number of mutations)	N (%)	Mutation Status (CGI)	Annotation on COSMIC or dbSNP
p.Ala58Val	1 (1.1)	Passenger	NA
p.Glu787Lys	1 (1.1)	Passenger	rs200378835
p.Lys238Asn	1 (1.1)	Polymorphism	rs1156281,COSM4144250
p.Pro696Leu	1 (1.1)	Polymorphism	rs10747493
ERBB3 (6)	6 (6.6)		
p.Ser1119Cys	2 (2.2)	Polymorphism	rs773123
p.Gly325Arg	1 (1.1)	Driver	COSM1935396
p.Gly582Ala	1 (1.1)	Driver	NA
p.Gly582Val	1 (1.1)	Driver	NA
p.Ser128Arg	1 (1.1)	Passenger	NA
NBN (6)	6 (6.6)		
p.Glu185Gln	2 (2.2)	Polymorphism	rs1805794,COSM3763443
c.*31delA_3'UTR	1 (1.1)	Not protein-affecting	NA
p.Ala46Val	1 (1.1)	Driver	NA
p.Arg551GlyfsTer8	1 (1.1)	Driver	COSM1458549
p.Pro672Leu	1 (1.1)	Passenger	NA
NOTCH3 (6)	6 (6.6)		
p.Ala1216Glu	1 (1.1)	Passenger	NA
p.Ala1802LeufsTer23	1 (1.1)	Passenger	NA
p.Ala2055Val	1 (1.1)	Passenger	rs769066713
p.Arg1837His	1 (1.1)	Passenger	rs138265894,COSM3529537,COSM3529538
p.Arg969Trp	1 (1.1)	Passenger	COSM1391046,COSM1391045
p.Lys2061Asn	1 (1.1)	Passenger	NA
RET (6)	5 (5.5)		
p.Gly691Ser	2 (2.2)	Polymorphism	rs1799939,COSM1666596,COSM1666597
p.Ala1046Val	1 (1.1)	Passenger	NA
p.Lys761Glu	1 (1.1)	Driver	NA
p.Thr48Met	1 (1.1)	Passenger	COSM1347795
p.Tyr864Cys	1 (1.1)	Driver	NA
ROS1 (6)	4 (4.4)		
p.Arg2039Cys	1 (1.1)	Driver	rs752085591,COSM6038976
p.Asp2213Asn	1 (1.1)	Polymorphism	rs529038,COSM3736400,COSM3736401,COSM150170

Gene (number of mutations)	N (%)	Mutation Status (CGI)	Annotation on COSMIC or dbSNP
p.Lys2228Gln	1 (1.1)	Polymorphism	rs529156,COSM3761458,COSM3761459,COSM150169
p.Pro556His	1 (1.1)	Driver	NA
p.Ser1031Phe	1 (1.1)	Driver	NA
p.Ser2229Cys	1 (1.1)	Polymorphism	rs619203,COSM3761456,COSM3761457,COSM150168
BRD3 (5)	5 (5.5)		
p.Pro24ArgfsTer24	2 (2.2)	Passenger	rs763134487,TMP_ESP_9_136918529_136918529,COSM1181066
p.Asn28Lys	1 (1.1)	Passenger	NA
p.Glu509_Lys510del	1 (1.1)	Passenger	rs569163807
p.Val473CysfsTer13	1 (1.1)	Passenger	NA
BRIP1 (5)	5 (5.5)		
p.Ser919Pro	3 (3.3)	Polymorphism	rs4986764
p.Arg106Cys	1 (1.1)	Passenger	rs587780247
p.Glu462Ter	1 (1.1)	Driver	NA
CHEK2 (5)	5 (5.5)		
c.*32C>A_3UTR	1 (1.1)	Not protein-affecting	NA
p.Asn359GlufsTer2	1 (1.1)	Driver	NA
p.Gly486Val	1 (1.1)	Driver	NA
p.Met534Thr	1 (1.1)	Passenger	NA
p.Ser207Asn	1 (1.1)	Driver	NA
FLT3 (5)	3 (3.3)		
p.Asn971LysfsTer43	1 (1.1)	Passenger	NA
p.Gln92Ter	1 (1.1)	Passenger	NA
p.Gln970Lys	1 (1.1)	Passenger	NA
p.Met665Val	1 (1.1)	Driver	NA
p.Thr227Met	1 (1.1)	Polymorphism	rs1933437,COSM5019176
BRCA2 (4)	4 (4.4)		
p.Asn372His	2 (2.2)	Neutral	rs144848,COSM3753646,COSM147663
p.Asn1784ThrfsTer7	1 (1.1)	Driver	COSM18607,COSM1639329
p.Glu182Gly	1 (1.1)	Driver	NA
CCND3 (4)	4 (4.4)		
p.Ser259Ala	4 (4.4)	Polymorphism	rs1051130,COSM451455
ERBB4 (4)	4 (4.4)		

Gene (number of mutations)	N (%)	Mutation Status (CGI)	Annotation on COSMIC or dbSNP
p.Ala287Val	1 (1.1)	Driver	NA
p.Ala4Val	1 (1.1)	Passenger	NA
p.Arg1273Trp	1 (1.1)	Driver	COSM1205584
p.Arg847His	1 (1.1)	Driver	COSM4681535
MSH6 (4)	4 (4.4)		
p.Phe1088SerfsTer2	3 (3.3)	Driver	rs770288143,COSM330655
p.Phe1088LeufsTer5	1 (1.1)	Driver	rs748452299,COSM308681
NOTCH2 (4)	4 (4.4)		
p.Asn853His	1 (1.1)	Passenger	NA
p.Gly1140Cys	1 (1.1)	Driver	NA
p.Gly1866Cys	1 (1.1)	Driver	NA
p.Tyr779Cys	1 (1.1)	Driver	NA
NRAS (4)	4 (4.4)		
p.Gly12Asp	2 (2.2)	Driver	rs121913237,COSM564
p.Gln61Lys	1 (1.1)	Driver	rs121913254,COSM580
p.Gly12Val	1 (1.1)	Driver	rs121913237,COSM566
PTCH1 (4)	4 (4.4)		
p.Pro1315Leu	3 (3.3)	Driver	rs357564,COSM1638394,COSM1638395,COSM1638396,COSM1638397
p.Val1435Met	1 (1.1)	Passenger	rs587778632,COSM3396768,COSM3396767,COSM3396770,COSM3396769
PTEN (4)	4 (4.4)		
p.Arg130Gln	1 (1.1)	Driver	rs121909229,COSM5033
p.Glu157GlyfsTer23	1 (1.1)	Driver	NA
p.Lys267ArgfsTer9	1 (1.1)	Driver	COSM30622
p.Lys332Ter	1 (1.1)	Driver	NA
AR (3)	3 (3.3)		
p.Arg841His	1 (1.1)	Driver	NA
p.Gln58Leu	1 (1.1)	Passenger	rs200185441,COSM376477
p.Leu57Gln	1 (1.1)	Neutral	rs78686797,COSM301687
AXL (3)	3 (3.3)		
p.Glu483Ter	1 (1.1)	Passenger	COSM1393945
p.Lys477del	1 (1.1)	Passenger	NA
p.Thr568Met	1 (1.1)	Passenger	rs749923407

Gene (number of mutations)	N (%)	Mutation Status (CGI)	Annotation on COSMIC or dbSNP
BRD2 (3)	3 (3.3)		
p.Ala474Val	1 (1.1)	Polymorphism	rs3918143
p.Arg281Trp	1 (1.1)	Driver	NA
p.Pro64ArgfsTer22	1 (1.1)	Driver	NA
CD22 (3)	3 (3.3)		
p.Pro342Ser	1 (1.1)	Passenger	rs781536221
p.Pro746His	1 (1.1)	Passenger	NA
p.Val344Ala	1 (1.1)	Passenger	NA
CDKN1A (3)	3 (3.3)		
p.Ser31Arg	2 (2.2)	Polymorphism	rs1801270,COSM3762017,COSM3762018
p.Pro4Leu	1 (1.1)	Passenger	rs4986866
CREBBP (3)	3 (3.3)		
p.Asn199Lys	1 (1.1)	Passenger	NA
p.Ile1084SerfsTer15	1 (1.1)	Driver	COSM1180829
p.Pro1279Leu	1 (1.1)	Passenger	rs749189606,COSM4060710
EGFR (3)	3 (3.3)		
p.Gly485Ser	1 (1.1)	Passenger	rs769434273
p.Pro848Leu	1 (1.1)	Driver	rs148934350,COSM22943
p.Thr725Met	1 (1.1)	Driver	rs767505234,COSM53264
ERBB2 (3)	2 (2.2)		
p.Pro1170Ala	2 (2.2)	Polymorphism	rs1058808
p.Ile655Val	1 (1.1)	Polymorphism	rs1136201,COSM4000121
FAM175A (3)	3 (3.3)		
p.Ala348Thr	2 (2.2)	Polymorphism	rs12642536,COSM3760790
p.Asp331Gly	1 (1.1)	Passenger	NA
KDR (3)	3 (3.3)		
p.Arg347His	1 (1.1)	Passenger	rs551579207,COSM327102
p.Asp39ThrfsTer38	1 (1.1)	Passenger	NA
p.Thr52Ser	1 (1.1)	Passenger	NA
MAP2K4 (3)	2 (2.2)		
p.Arg281Ter	1 (1.1)	Driver	COSM218507
p.Arg287His	1 (1.1)	Driver	COSM180952

Gene (number of mutations)	N (%)	Mutation Status (CGI)	Annotation on COSMIC or dbSNP
p.Val398Ala	1 (1.1)	Passenger	NA
MET (3)	3 (3.3)		
p.Arg1188Ter	1 (1.1)	Passenger	COSM1214928
p.Met362Thr	1 (1.1)	Passenger	rs77523018
p.Thr1011Ala	1 (1.1)	Passenger	rs587778441
MLH1 (3)	3 (3.3)		
p.Ile219Val	2 (2.2)	Neutral	rs1799977,MMR_c.655A>G,COSM1131469
p.Arg100Ter	1 (1.1)	Driver	rs63751221,COSM29727
MTOR (3)	3 (3.3)		
p.Gly1846Ser	1 (1.1)	Passenger	NA
p.Ser2215Phe	1 (1.1)	Driver	COSM1686998
p.Thr2444Met	1 (1.1)	Driver	rs769849522
MUTYH (3)	3 (3.3)		
p.Gln335His	2 (2.2)	Polymorphism	rs3219489,COSM3751252
p.Val22Met	1 (1.1)	Polymorphism	rs3219484
NFKBIA (3)	3 (3.3)		
c.*2C>T_3'UTR	3 (3.3)	Polymorphism	rs8904
STAT5B (3)	3 (3.3)		
p.Arg200Gln	1 (1.1)	Driver	rs779220548
p.Lys164SerfsTer17	1 (1.1)	Driver	COSM1383347
p.Lys583AsnfsTer16	1 (1.1)	Driver	COSM1383340
TERT (3)	3 (3.3)		
p.Ala855Val	1 (1.1)	Passenger	rs775014633,COSM1228934,COSM1228935
p.Val1035Ile	1 (1.1)	Passenger	rs374968697
p.Val826Ile	1 (1.1)	Passenger	NA
ARAF (2)	2 (2.2)		
p.Arg255GlyfsTer37	1 (1.1)	Passenger	COSM1468139
p.Arg603Cys	1 (1.1)	Driver	COSM2965923
AURKA (2)	2 (2.2)		
p.Ile57Val	1 (1.1)	Polymorphism	rs1047972
p.Phe31Ile	1 (1.1)	Passenger	rs2273535,COSM3736282
AURKB (2)	2 (2.2)		

Gene (number of mutations)	N (%)	Mutation Status (CGI)	Annotation on COSMIC or dbSNP
p.Met298Thr	2 (2.2)	Polymorphism	rs1059476,COSM4130880,COSM4130881
CDKN2A (2)	2 (2.2)		
p.Arg58Ter	1 (1.1)	Driver	rs121913387,COSM12473,COSM99730,COSM99731,COSM1624870
p.Met154IlefsTer76	1 (1.1)	Not protein-affecting	NA
CRKL (2)	2 (2.2)		
p.Arg164Trp	1 (1.1)	Passenger	rs760471921
p.Pro67Thr	1 (1.1)	Passenger	NA
HDAC4 (2)	2 (2.2)		
c.2989-2A>G_p.X997_splice	1 (1.1)	Passenger	COSM4092775
p.Arg283Cys	1 (1.1)	Passenger	COSM1018638
HDAC7 (2)	2 (2.2)		
p.Arg106Trp	1 (1.1)	Passenger	rs779766353
p.Glu958del	1 (1.1)	Passenger	rs769122679
IDH1 (2)	2 (2.2)		
c.-16-1G>T_splice site	1 (1.1)	Passenger	NA
p.Val178Ile	1 (1.1)	Polymorphism	rs34218846,COSM97131
JAK1 (2)	2 (2.2)		
p.Ala449Thr	1 (1.1)	Driver	rs765489448,COSM1343910
p.Asn339IlefsTer3	1 (1.1)	Driver	COSM1343915
JAK3 (2)	2 (2.2)		
p.Asn419Thr	1 (1.1)	Passenger	NA
p.Glu552Lys	1 (1.1)	Passenger	NA
MDM2 (2)	2 (2.2)		
p.Asn131Ser	1 (1.1)	Passenger	rs777635548
p.Cys455Tyr	1 (1.1)	Passenger	NA
MRE11A (2)	2 (2.2)		
p.Arg572Ter	1 (1.1)	Driver	rs137852761,MRE11Abase_D0061:g.47557C>T,COSM378985,COSM378984
p.Gln677Glu	1 (1.1)	Passenger	NA
MS4A1 (2)	2 (2.2)		
p.Glu237AsnfsTer6	1 (1.1)	Passenger	rs779922206
p.Trp216Ter	1 (1.1)	Passenger	NA
NT5C2 (2)	2 (2.2)		

Gene (number of mutations)	N (%)	Mutation Status (CGI)	Annotation on COSMIC or dbSNP
p.Phe450LeufsTer5	1 (1.1)	Driver	NA
p.Thr3Ala	1 (1.1)	Polymorphism	rs10883841
PIK3CG (2)	2 (2.2)		
p.Ala85Val	1 (1.1)	Passenger	NA
p.Thr857Ala	1 (1.1)	Polymorphism	rs28763991,COSM3762311
PMS2 (2)	2 (2.2)		
p.Pro470Ser	1 (1.1)	Neutral	rs1805321,MMR_c.1408C>T,COSM3736445
p.Val549Met	1 (1.1)	Passenger	NA
RRM1 (2)	2 (2.2)		
p.Arg499His	1 (1.1)	Passenger	COSM263457
p.Pro639Ser	1 (1.1)	Passenger	NA
RUNX1 (2)	2 (2.2)		
c.-2C>T_5'UTR	1 (1.1)	Not protein-affecting	rs375033964
p.Ser362Leu	1 (1.1)	Passenger	COSM4170266
SOX2 (2)	2 (2.2)		
p.His162Tyr	1 (1.1)	Passenger	NA
p.Lys95Glu	1 (1.1)	Passenger	NA
SRC (2)	2 (2.2)		
p.Glu100Lys	1 (1.1)	Passenger	NA
p.Thr182Ala	1 (1.1)	Passenger	NA
TSC1 (2)	2 (2.2)		
p.Lys587Arg	1 (1.1)	Polymorphism	rs118203576
p.Ser1111Asn	1 (1.1)	Passenger	NA
ABL1 (1)	1 (1.1)		
p.Asn72ThrfsTer28	1 (1.1)	Passenger	NA
AKT1 (1)	1 (1.1)		
p.Glu17Lys	1 (1.1)	Driver	rs121434592,COSM33765
AXIN1 (1)	1 (1.1)		
p.Val742Ile	1 (1.1)	Passenger	rs374579262
BRD4 (1)	1 (1.1)		
p.Gly732Arg	1 (1.1)	Passenger	NA
CCNE1 (1)	1 (1.1)		

Gene (number of mutations)	N (%)	Mutation Status (CGI)	Annotation on COSMIC or dbSNP
p.Met336Ile	1 (1.1)	Passenger	COSM4076587
CDH1 (1)	1 (1.1)		
p.Arg224His	1 (1.1)	Passenger	rs201511530,COSM972782
CDK12 (1)	1 (1.1)		
p.Glu182del	1 (1.1)	Passenger	NA
DDR2 (1)	1 (1.1)		
p.Arg473His	1 (1.1)	Passenger	rs775098890,COSM1648427,COSM530257,COSM1648428
EPHA7 (1)	1 (1.1)		
p.Glu536Ter	1 (1.1)	Passenger	NA
FGFR3 (1)	1 (1.1)		
p.Lys405GlnfsTer93	1 (1.1)	Passenger	rs775260779
GATA2 (1)	1 (1.1)		
p.Ala478Thr	1 (1.1)	Driver	rs780263343
HGF (1)	1 (1.1)		
p.Arg494Ter	1 (1.1)	Passenger	NA
HRAS (1)	1 (1.1)		
p.Glu37Gln	1 (1.1)	Passenger	NA
IDH2 (1)	1 (1.1)		
p.Arg172Ser	1 (1.1)	Driver	COSM133672
IGF1R (1)	1 (1.1)		
p.Ser522Thr	1 (1.1)	Passenger	NA
MAP2K1 (1)	1 (1.1)		
c.-31dupC_5'UTR	1 (1.1)	Polymorphism	rs142027715
MAPK1 (1)	1 (1.1)		
p.Met333Val	1 (1.1)	Passenger	NA
MPL (1)	1 (1.1)		
p.Leu79Met	1 (1.1)	Driver	NA
MSH2 (1)	1 (1.1)		
p.Asn127Ser	1 (1.1)	Passenger	rs17217772,MMR_c.380A>G
MYC (1)	1 (1.1)		
p.Asn26Ser	1 (1.1)	Polymorphism	rs4645959
MYD88 (1)	1 (1.1)		

Gene (number of mutations)	N (%)	Mutation Status (CGI)	Annotation on COSMIC or dbSNP
p.Pro182His	1 (1.1)	Passenger	NA
NTRK1 (1)	1 (1.1)		
p.His297Tyr	1 (1.1)	Driver	NA
PALB2 (1)	1 (1.1)		
p.Arg1086Ter	1 (1.1)	Driver	rs587776527
PDGFRA (1)	1 (1.1)		
p.Arg500Ter	1 (1.1)	Passenger	NA
PDGFRB (1)	1 (1.1)		
p.Leu1086Pro	1 (1.1)	Passenger	NA
PIK3CB (1)	1 (1.1)		
p.Glu634Ter	1 (1.1)	Passenger	NA
RAF1 (1)	1 (1.1)		
p.Pro643Leu	1 (1.1)	Passenger	NA
RICTOR (1)	1 (1.1)		
p.Ser837Phe	1 (1.1)	Polymorphism	rs2043112,COSM4003526,COSM4003525
SMO (1)	1 (1.1)		
p.Arg482Cys	1 (1.1)	Driver	rs755295928,COSM3878026
TSC2 (1)	1 (1.1)		
p.Phe1510del	1 (1.1)	Passenger	rs386833415
VEGFA (1)	1 (1.1)		
p.Leu192Ile	1 (1.1)	Passenger	NA
XPO1 (1)	1 (1.1)		
p.Asp891His	1 (1.1)	Passenger	NA

N = number of patients with the mutation.

Mutation status was determined by Cancer Genome Interpreter (CGI) and olny mutations assigned as Driver was considered in the analyses

Table S2. Exclusive genes found mutated in microsatellite instable and stable tumors

MSI-positive		MSI-negative	
Gene	(N)	Gene	(N)
AKT1	(1)	*ALK*	(1)
BRCA1	(1)	*AR*	(1)
CREBBP	(1)	*ARAF*	(1)
EGFR	(2)	*BARD1*	(1)
FLT3	(1)	*BRIP1*	(1)
JAK1	(2)	*CDKN2A*	(1)
MAP3K1	(2)	*ERBB3*	(3)
MLH1	(1)	*ERBB4*	(3)
MPL	(1)	*FLT4*	(1)
MRE11A	(1)	*GATA2*	(1)
MSH6	(4)	*IDH2*	(1)
NBN	(2)	*MAP2K4*	(2)
NOTCH2	(3)	*NRAS*	(4)
NT5C2	(1)	*NTRK1*	(1)
PALB2	(1)		
RET	(2)		
SMO	(1)		
STAT5B	(3)		
TGFRBR2	(9)		

Table S3. Mutation profile by the proportion of African, European, Asian and Native American ancestry

	African Ancestry				European Ancestry				Asian Ancestry				Native American Ancestry			
	Low (N= 30)	Intermediate (N=30)	High (N=29)		Low (N=30)	Intermediate (N=30)	High (N-29)		Low (N=30)	Intermediate (N=30)	High (N-29)		Low (N=30)	Intermediate (N=30)	High (N-29)	
	N (%)	N (%)	N (%)	*P* value	N (%)	N (%)	N (%)	*P* value	N (%)	N (%)	N (%)	*P* value	N (%)	N (%)	N (%)	*P* value
APC	22 (73.3)	20 (66.7)	21 (72.4)	0.876*	20 (66.7)	23 (76.7)	20 (69.0)	0.705*	21 (67.7)	22 (75.9)	20 (69.0)	0.838*	22 (68.8)	24 (85.7)	17 (58.6)	0.069**
TP53	17 (56.7)	16 (53.3)	18 (62.1)	0.830*	16 (53.3)	16 (53.3)	19 (65.5)	0.551*	20 (64.5)	15 (51.7)	16 (55.2)	0.637*	23 (71.9)	19 (67.9)	9 (31.0)	0.002*
KRAS	15 (50.0)	20 (66.7)	13 (44.8)	0.213*	17 (56.7)	14 (46.7)	17 (58.6)	0.644*	14 (45.2)	20 (69.0)	14 (48.3)	0.156*	18 (56.3)	15 (53.6)	15 (51.7)	0.964*
PIK3CA	3 (10.0)	7 (23.3)	4 (13.8)	0.386**	6 (20.0)	6 (20.0)	2 (6.9)	0.289**	4 (12.9)	5 (17.2)	5 (17.2)	0.874**	2 (6.3)	3 (10.7)	9 (31.0)	0.024**
FBXW7	4 (13.3)	2 (6.7)	4 (13.8)	0.703**	4 (13.3)	3 (10.0)	3 (10.3)	>0.999**	3 (9.7)	5 (17.2)	2 (6.9)	0.494**	4 (12.5)	3 (10.7)	3 (10.3)	>0.999**
NF1	0 (0.0)	3 (10.0)	5 (17.2)	0.045**	4 (13.3)	4 (13.3)	0 (0.0)	0.119**	1 (3.2)	1 (3.4)	6 (20.7)	0.037**	2 (6.3)	2 (7.1)	4 (13.8)	0.647**
TGFBR2	1 (3.3)	4 (13.3)	3 (10.3)	0.430**	2 (6.7)	4 (13.3)	2 (6.9)	0.722**	3 (9.7)	3 (10.3)	2 (6.9)	>0.999**	4 (12.5)	1 (3.6)	3 (10.3)	0.526**
ATM	3 (10.0)	1 (3.3)	2 (6.9)	0.692**	2 (6.7)	1 (3.3)	3 (10.3)	0.521**	3 (9.7)	1 (3.4)	2 (6.9)	0.868**	4 (12.5)	0 (0.0)	2 (6.9)	0.182**
BRAF	1 (3.3)	1 (3.3)	6 (20.7)	0.031**	5 (16.7)	2 (6.7)	1 (3.4)	0.263**	3 (9.7)	1 (3.4)	4 (13.8)	0.428**	2 (6.3)	2 (7.1)	4 (13.8)	0.647**
BLM	1 (3.3)	4 (13.3)	2 (6.9)	0.393**	2 (6.7)	4 (13.3)	1 (3.4)	0.493**	2 (6.5)	2 (6.9)	3 (10.3)	0.892**	3 (9.4)	1 (3.6)	3 (10.3)	0.694**
MSH6	1 (3.3)	2 (6.7)	1 (3.4)	>0.999**	0 (0.0)	3 (10.0)	1 (3.4)	0.218**	2 (6.5)	1 (3.4)	1 (3.4)	>0.999**	2 (6.3)	1 (3.6)	1 (3.4)	>0.999**
NOTCH1	0 (0.0)	2 (6.7)	1 (3.4)	0.540**	2 (6.7)	1 (3.3)	0 (0.0)	0.770**	0 (0.0)	2 (6.9)	1 (3.4)	0.311**	0 (0.0)	1 (3.6)	2 (6.9)	0.301**
NRAS	1 (3.3)	2 (6.7)	1 (3.4)	>0.999**	1 (3.3)	2 (6.7)	1 (3.4)	>0.999**	1 (3.2)	1 (3.4)	2 (6.9)	0.840**	2 (6.3)	1 (3.6)	1 (3.4)	>0.999**
PTEN	0 (0.0)	1 (3.3)	3 (10.3)	0.122**	3 (10.0)	1 (3.3)	0 (0.0)	0.318**	1 (3.2)	0 (0.0)	3 (10.3)	0.212**	2 (6.3)	0 (0.0)	2 (6.9)	0.542**
CHEK2	0 (0.0)	1 (3.3)	2 (6.9)	0.318**	2 (6.7)	1 (3.3)	0 (0.0)	0.770**	0 (0.0)	2 (6.9)	1 (3.4)	0.311**	0 (0.0)	1 (3.6)	2 (6.9)	0.301**
ERBB3	1 (3.3)	1 (3.3)	1 (3.4)	>0.999**	1 (3.3)	1 (3.3)	1 (3.4)	>0.999**	1 (3.2)	0 (0.0)	2 (6.9)	0.533**	1 (3.1)	0 (0.0)	2 (6.9)	0.522**
ERBB4	1 (3.3)	0 (0.0)	2 (6.9)	0.318**	2 (6.7)	1 (3.3)	0 (0.0)	0.770**	0 (0.0)	0 (0.0)	3 (10.3)	0.064**	1 (3.1)	0 (0.0)	2 (6.9)	0.522**
NOTCH2	0 (0.0)	1 (3.3)	2 (6.9)	0.318**	1 (3.3)	2 (6.7)	0 (0.0)	0.770**	1 (3.2)	1 (3.4)	1 (3.4)	>0.999**	1 (3.1)	2 (7.1)	0 (0.0)	0.408**
ROS1	0 (0.0)	1 (3.3)	2 (6.9)	0.318**	2 (6.7)	1 (3.3)	0 (0.0)	0.770**	0 (0.0)	0 (0.0)	3 (10.3)	0.064**	0 (0.0)	1 (3.6)	2 (6.9)	0.301**
STAT5B	0 (0.0)	2 (6.7)	1 (3.4)	0.540**	1 (3.3)	1 (3.3)	1 (3.4)	>0.999**	1 (3.2)	2 (6.9)	0 (0.0)	0.533**	1 (3.1)	1 (3.6)	1 (3.4)	>0.999**

* Chi-squared test; ** Fisher Exact Test

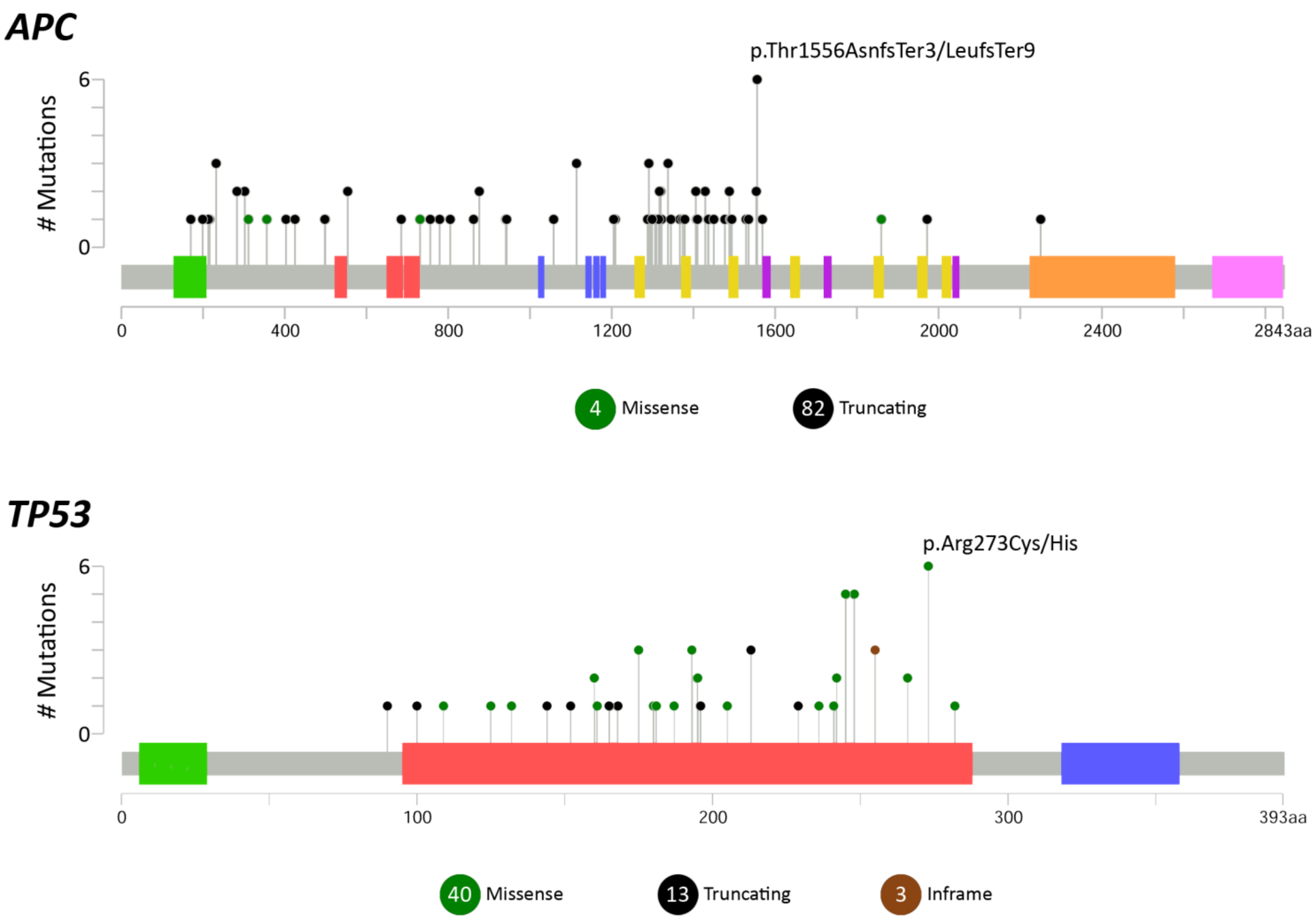

Figure S1. Mutation mapper of the *APC* and *TP53* genes. While *APC* showed mostly truncating mutations spread throughout the gene, the *TP53* gene showed mutations clustered on the DNA-binding domain.

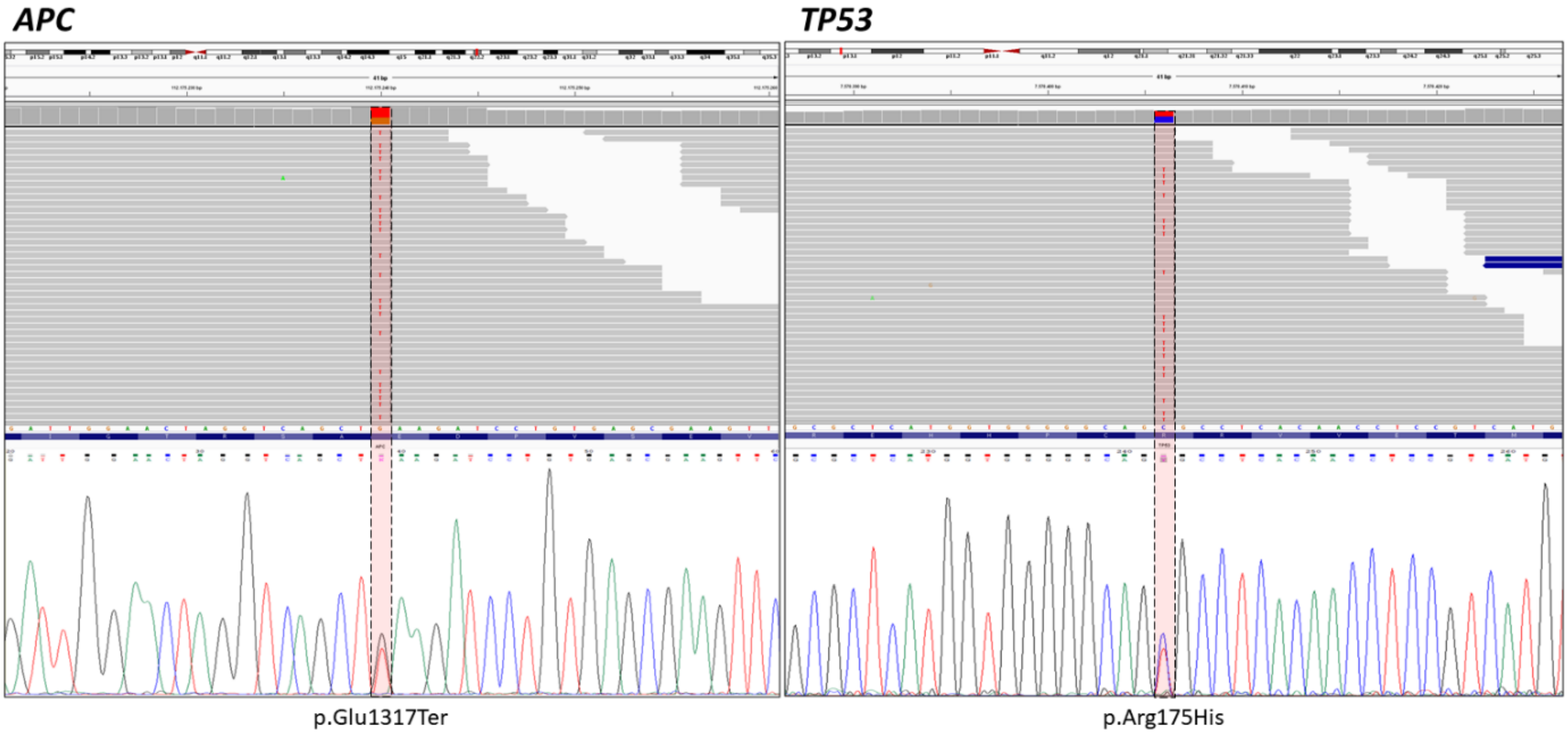

Figure S2. Mutation validation using Sanger sequencing of genes *APC* (left) and *TP53* (right). The figure shows the same mutation (red box) observed by NGS data in Integrative Genomics Viewer (top panel) and Sanger sequencing (bottom panel).

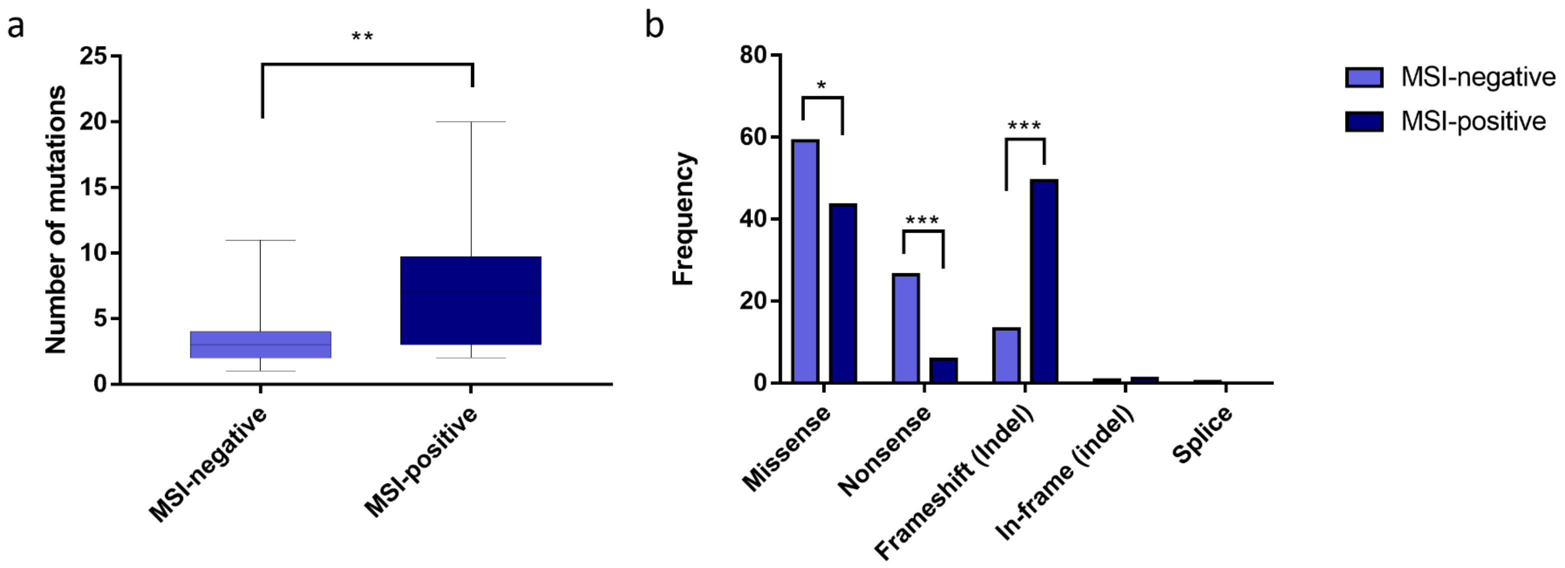

Figure S3. Differences between tumors with (MSI-positive) and without (MSI-negative) microsatellite instability. The Mann-Whitney test showed that MSI tumors had a higher number of mutations (a), and the chi-square test showed differences between mutation types (b). * $p<0.05$; ** $p<0.005$; *** $p<0.001$.

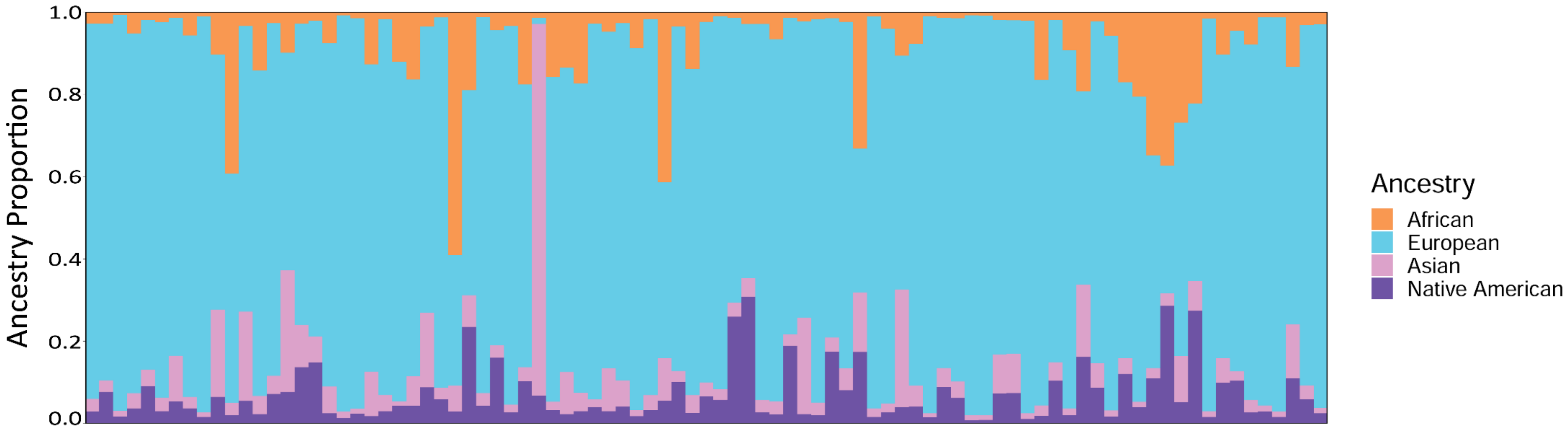

Figure S4. Individual ancestry estimated by 46 autosomal ancestry informative markers (AIMs), consisting of insertion and deletion polymorphisms and using the genetic data of the panel of diversity Human Genome Diversity Project – center d'Etude du Polymorphisme Humain (HGDP-CEPH).
